# The Maize ZmbHLH118 Transcription Factor Regulates Vacuolar Nitrate Loading by the NO_3_^−^ Transporter ZmCLCa

**DOI:** 10.1002/advs.202520219

**Published:** 2026-02-21

**Authors:** Chaonan Zhang, Elsa Demes‐Causse, Xujian Li, Zhenhui Guo, Hanshu Zhao, Huairong Cao, Yajing Song, Lijun Mu, Kaikai Zhang, Jing Zhang, Zhongtao Jia, Lixing Yuan, Alexis De Angeli, Jingbo Zhang

**Affiliations:** ^1^ State Key Laboratory of Nutrient Use and Management College of Resources and Environmental Sciences National Academy of Agriculture Green Development China Agricultural University Beijing China; ^2^ IPSiM CNRS INRAE Institut Agro Université Montpellier Montpellier France; ^3^ State Key Laboratory of Plant Environmental Resilience College of Biological Sciences Center For Crop Functional Genomics and Molecular Breeding Frontiers Science Center For Molecular Design Breeding (MOE) China Agricultural University Beijing China; ^4^ Sanya Institute of China Agricultural University Sanya China

**Keywords:** maize, nitrate, vacuole, ZmbHLH118, ZmCLCa

## Abstract

Nitrate (NO_3_^−^) is a major nutrient promoting plant growth and crop yield. Vacuolar nitrate storage is essential for nitrate acquisition and remobilization within the plant. However, the transcriptional regulation of transporters facilitating nitrate influx into the vacuole remains unclear. Here, we identified a bHLH transcription factor, ZmbHLH118, that negatively regulates the expression of *ZmCLCa*, thereby modulating nitrate vacuolar loading and uptake in maize (*Zea mays*). ZmbHLH118 overexpression and ZmCLCa loss‐of‐function in maize impaired root NO_3_^−^ uptake, and further reduced nitrate content and plant growth. Moreover, ZmbHLH118 directly binds to the promoter and inhibits the expression of *ZmCLCa*, which encodes a tonoplast‐localized nitrate transporter. Electrophysiological analysis showed that ZmCLCa mediates NO_3_^−^ fluxes across the vacuolar membrane, indicating that ZmCLCa‐mediated NO_3_^−^ influx is required for vacuolar nitrate storage. Our data identify the ZmbHLH118 as a molecular actor of the transcriptional regulation of *ZmCLCa* in response to extracellular nitrate. This provides insights into the regulatory mechanisms acting upstream of the CLCa‐mediated vacuolar nitrate transport. Finally, field assays showed that the regulation mechanisms of vacuolar nitrate affect maize growth and grain yield, highlighting their value for nitrogen use efficiency improvement in crop plants.

## Graphical abstract

In maize, the bHLH transcription factor ZmbHLH118 directly binds to the promoter of *ZmCLCa* and inhibits its expression. Tonoplast‐localized ZmCLCa mediates NO_3_^−^ influx into the vacuole to regulate intracellular NO_3_^−^ homeostasis, modulating nitrate uptake and metabolism, plant growth, and grain yield. ZmbHLH118‐ZmCLCa module provides insights into the regulatory mechanisms in CLCa‐mediated nitrate transport.

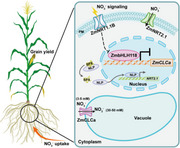

## 1 Introduction

Nitrogen (N) is essential for plants to synthesize amino acids, nucleotides, and proteins, and further regulate plant growth and development [1, 2]. For most terrestrial crops, nitrate (NO_3_^−^) is the dominant nitrogen source for crop production [3, 4]. Although the application of chemical nitrogen fertilizers dramatically improved crop yield, it also caused serious environmental pollution [5, 6, 7, 8]. Therefore, improving crop nitrogen use efficiency (NUE) is crucial for reducing chemical fertilizer consumption and promoting sustainable agriculture [9, 10, 11].

Plants evolved complex networks to regulate nitrate uptake, translocation, metabolism, and distribution to maintain cellular nitrate homeostasis [12, 13, 14]. Within the cell, nitrate is assimilated into organic molecules to maintain plant growth and development [15, 16, 17, 18]. However, to prevent cytoplasmic toxicity, excess nitrate is stored in the vacuole and can be remobilized for utilization under N‐limited conditions [19, 20, 21]. The vacuole, a multifunctional and complex compartment [22], occupies most of the cellular space and stores around 90% of intracellular nitrate [19, 23, 24]. Tonoplast‐localized nitrate transporters and channels are required to keep vacuolar nitrate accumulation and fine‐tune cytosolic concentration [25].

In the past few decades, many vacuolar nitrate transporters have been identified and characterized [26, 27, 28, 29, 30, 31], among which AtCLCa is the most well‐studied [24, 32, 33, 34, 35, 36, 37, 38, 39, 40, 41]. AtCLCa belongs to the CLC family, which is involved in osmoregulation and turgor pressure, stomatal movements, metal tolerance, and nitrate absorption in plants [36]. In *Arabidopsis thaliana*, four CLCs, AtCLCa, AtCLCb, AtCLCc, and AtCLCg, are localized in the tonoplast [39]. AtCLCa is a 2NO_3_^−^/1H^+^ antiporter and mediates NO_3_^−^ transport into the vacuole, modulating intracellular nitrate and pH homeostasis in *Arabidopsis* [24, 32, 33]. The nitrate transport activity of AtCLCa is regulated by the AMP/ATP ratio, which is related to the nitrate metabolism in plants [34]. The nitrate selectivity of AtCLCa is conferred by a single amino acid residue, Proline 160, which does not affect the anion–proton coupling [38]. The mutant *AtCLCa_P160S_* loses nitrate selectivity and, when expressed in *clca* plants, it fails to complement the reduced nitrate accumulation phenotype [38, 40]. The mutation of *AtCLCa‐E203A* converts AtCLCa into a NO_3_^−^ channel [35], resulting in a growth deficit while increasing the NUE through greater allocation of assimilated nitrogen to the seeds [41]. In *Arabidopsis*, AtCLCb is also a nitrate exchanger, but no nitrate content decrease was detected in *clcb* knock‐out mutant plants [42]. Notably, *AtCLCa* is transcriptionally regulated by nitrate [32], but its upstream regulatory mechanisms remain unknown. Overall, relative to *Arabidopsis*, vacuolar nitrate transport has been far less studied in crop plants, and whether they share similar regulatory mechanisms remains unclear.

The existence of nitrate‐sensing mechanisms is well documented, and nitrate signaling pathways have been extensively characterized over the past few decades [43, 44, 45, 46, 47, 48, 49]. NRT1.1‐NLPs cascade forms the core nitrate signaling pathway [50, 51, 52, 53]. At the plasma membrane, NRT1.1 functions as a dual‐affinity NO_3_^−^ transporter and a sensor, mediating NO_3_^−^ uptake and translocation in *Arabidopsis* and crop plants [50, 54, 55, 56]. Phosphorylation of the Threonine 101 (T101) by CIPK23 converts NRT1.1 from low‐ to high‐affinity [57]. Together with CNGC15, NRT1.1 triggers a rapid cytoplasmic Ca^2+^ rise, leading to the phosphorylation of NLP7 by calcium‐dependent protein kinases (CPKs) [52]. NLP7 is an intracellular sensor that directly binds NO_3_^−^ and regulates gene expression to control NO_3_^−^ uptake and metabolism [53, 58]. The OsNRT1.1B‐OsSPX4‐OsNLP3 module transduces nitrate signaling from the plasma membrane to the nucleus, promoting NUE in rice [50, 51]. Surprisingly, a recent study showed that OsNRT1.1B is also an abscisic acid (ABA) receptor and modulates nitrate‐mediated ABA signaling in response to environmental nutrient fluctuations [59]. In maize, the plasma membrane‐localized dual‐affinity nitrate transporter ZmNRT1.1B [60] promotes nitrate‐induced nuclear translocation of ZmNLP3.1, enhancing grain yield and NUE [61]. Another maize transcription factor, ZmNLP3.2, regulates root growth via the ZmNLP3.2‐ZmARF19‐ZmAux/IAA14 module [62]. Although the nitrate signaling pathways are well understood in plants, whether and how NRT1.1‐mediated nitrate signaling regulates vacuolar membrane NO_3_^−^ transport remains largely elusive.

The basic helix‐loop‐helix (bHLH) family is one of the largest transcription factor families in plants, with crucial roles in diverse physiological processes [63, 64, 65, 66, 67, 68, 69, 70, 71, 72, 73, 74, 75, 76, 77, 78]. bHLHs bind to a consistent hexanucleotide E‐box (5′‐CANNTG‐3′) at the promoter region of the target gene, modulating gene expression [79]. In maize, the bHLH family consists of more than two hundred members [80], and several bHLHs have been identified [66, 67, 68, 69, 70, 71, 73]. COOL1 negatively regulates the expression of cold‐response genes and modulates cold tolerance in maize [66]. Jasmonic acid regulates ZmbHLH154 and ZmIBH1 by attenuating brassinosteroid (BR) signaling, modulating the expression of cell wall‐related genes involved in maize internode elongation [73]. Moreover, ZmbHLH121 positively regulates the formation of root cortical aerenchyma, promoting water and nutrient acquisition in maize plants [71]. *Ms23*, *Ms32*, *ZmbHLH122*, and *ZmbHLH51* are sequentially expressed in the tapetum, thereby ensuring the establishment of male fertility and anther development in maize through mutual regulation or heterodimer formation [67, 68, 69, 70]. Notably, bHLH transcription factors were reported to function in the nitrate signaling pathway via interacting with NLP7 [74, 77]. In *Arabidopsis*, the bHLH protein HBI1 functions downstream of NLP6 and NLP7 to positively regulate nitrate signaling through regulation of ROS homeostasis [74]. In wheat, TabHLH489 directly interacts with TaNLP7‐3A and inhibits its transcriptional activity in nitrate signaling [77]. Moreover, TabHLH489 promotes ROS accumulation, which in turn reduces the nuclear localization of TaNLP7‐3A and compromises its ability to regulate nitrogen‐responsive gene expression [77]. However, the understanding of maize bHLHs in nitrate signaling is limited, and their roles in regulating vacuolar anion transporters and channels remain unclear.

In this study, we revealed that a bHLH transcription factor, ZmbHLH118, negatively regulates nitrate content and plant growth in maize by directly binding to the *ZmCLCa* promoter to inhibit its expression. *ZmCLCa* encodes a tonoplast‐localized nitrate transporter that positively regulates the nitrate content and maize plant growth. Electrophysiological assay showed that ZmCLCa mediates nitrate anion influx into the vacuole for storage. Both overexpression of ZmbHLH118 and function loss of ZmCLCa impair nitrate uptake in maize roots, resulting in decreased nitrate content and inhibited plant growth. Moreover, ZmbHLH118 and ZmCLCa play important roles in regulating the grain yield in the field and NUE. Collectively, our findings uncover a ZmbHLH118‐ZmCLCa module that regulates vacuolar nitrate transport and intracellular nitrate homeostasis in maize, providing new insights into the regulation of CLCa‐mediated NO_3_^−^ transport.

## 2 Results

### 2.1 ZmbHLH118 Negatively Regulates Maize Growth and Nitrate Content

To explore the regulatory roles of transcription factors (TF) in maize (*Zea mays*) plants, we screened a transgenic population harboring overexpression of maize TF genes under the *Ubiquitin* promoter. Grown under low nitrate (LN, 0.04 mm KNO_3_) and normal nitrate (NN, 4 mm KNO_3_) conditions, two independent maize transgenic lines were identified, in which a bHLH transcription factor coding gene, *ZmbHLH118* (GRMZM2G061906), is overexpressed (Figure S1). These two independent overexpression lines (ZmbHLH118‐OE1 and ZmbHLH118‐OE2) were used for further studies.

**FIGURE 1:**
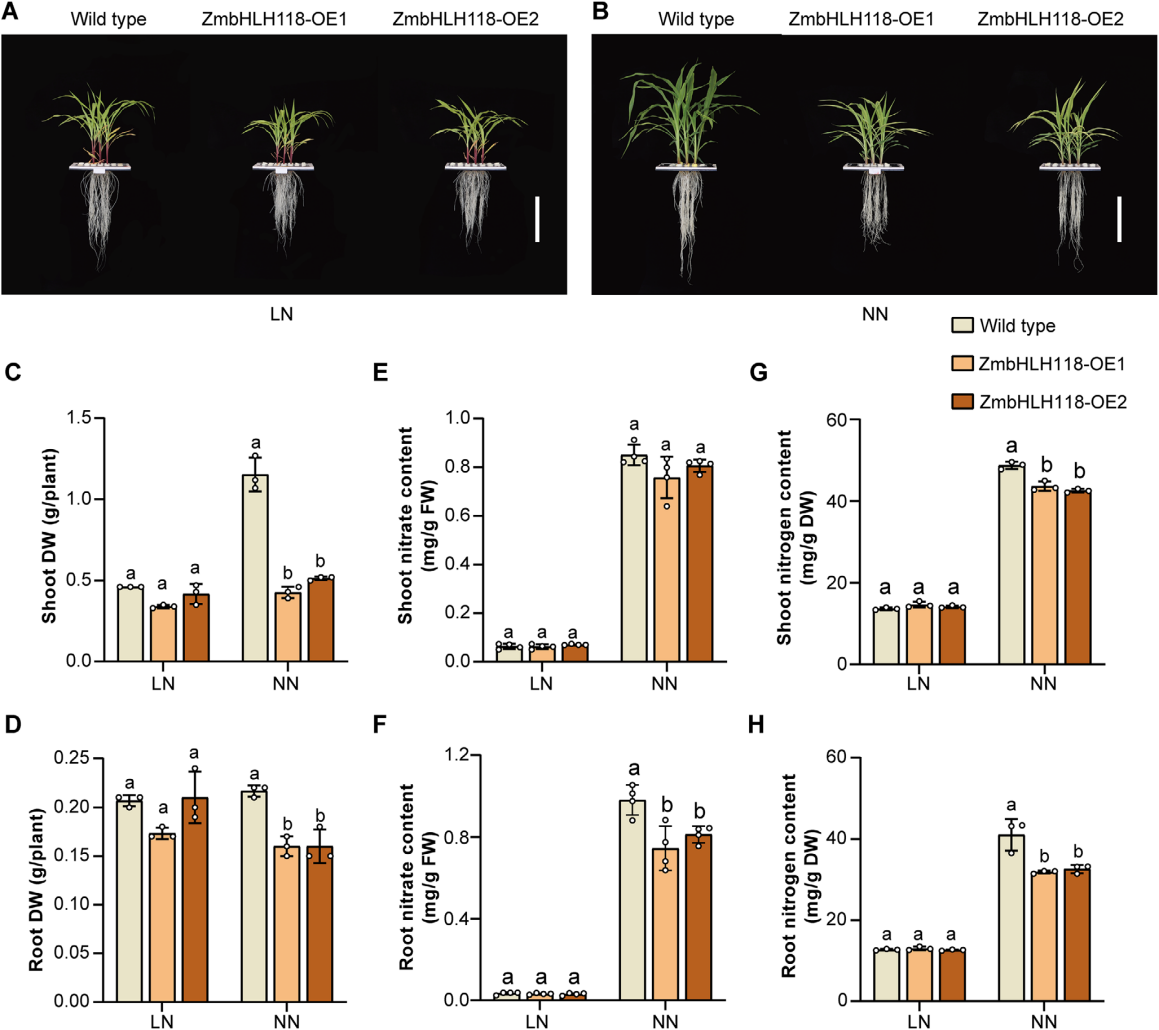
ZmbHLH118 negatively regulates maize growth and nitrate content. Three‐week‐old ZmbHLH118 overexpressing (ZmbHLH118‐OE) and wild‐type maize plants growth under (A) Low Nitrate (LN, 0.04 mm KNO_3_) and (B) Normal Nitrate (NN, 4 mm KNO_3_) conditions. (C, D) The biomass, (E, F) nitrate concentration, and (G, H) nitrogen concentration in shoot and root tissue of ZmbHLH118 overexpressing and wild‐type maize plants grown in the indicated nitrate conditions. DW, dry weight; FW, fresh weight. (A and B) Scale bars, 20 cm. Data in (C–H) are means ± SD (*n* = 3–4 biological replicates). Statistical significance was determined using one‐way ANOVA followed by Tukey's multiple comparison test. Different letters represent a significant difference at *p* < 0.05.

Grown under NN and LN conditions, observations of 3‐week‐old maize seedling growth phenotypes indicated that overexpression of ZmbHLH118 significantly inhibits plant growth under NN but not LN conditions (Figure 1A,B). Dry weight measurements revealed that ZmbHLH118‐OE1 and ZmbHLH118‐OE2 seedlings had significantly lower biomass than wild‐type plants at NN conditions (Figure 1C,D). Specifically, shoot dry weight decreased by 63% and 55%, and root dry weight decreased by 26% in ZmbHLH118‐OE1 and ZmbHLH118‐OE2 plants, respectively (Figure 1C,D). Additionally, nitrate and nitrogen measurements were conducted, showing that ZmbHLH118 overexpressing plants (ZmbHLH118‐OE1 and ZmbHLH118‐OE2) exhibited lower contents of both nitrate and nitrogen (Figure 1E–H). Notably, lower contents of nitrate and nitrogen in ZmbHLH118 overexpressing plants were more pronounced in the roots (Figure 1F,H). For instance, compared to the wild type, no significant changes in nitrate content in the shoots were observed (Figure 1E), while 24% and 17% decreases were observed in the roots of ZmbHLH118‐OE1 and ZmbHLH118‐OE2 plants, respectively (Figure 1F). Similarly, 10% and 13% decrease of nitrogen content in the shoots (Figure 1G) with 22% and 21% decrease in the roots (Figure 1H) were detected in ZmbHLH118‐OE1 and ZmbHLH118‐OE2 maize plants, respectively.

To further investigate the roles of ZmbHLH118 in maize growth and nitrate utilization, we obtained one EMS‐induced mutagenesis mutant, *zmbhlh118‐ems*, which produced a truncated ZmbHLH118 protein with a nucleotide base substitution (from C to T) at the position of residue 123 in the fourth exon (Figure S2A). We conducted similar measurements as described above and found that *zmbhlh118‐ems* mutant plants exhibited better growth (Figure S2B,C) and significantly higher biomass than the wild type under NN conditions (Figure S2D,E). However, no significant differences in nitrate (Figure S2F,G) and nitrogen content (Figure S2H,I) were detected between *zmbhlh118‐ems* and the wild‐type plants.

Taken together, our data indicated that ZmbHLH118 negatively regulates plant growth and nitrate content in maize.

### 2.2 ZmbHLH118 has Transcriptional Inhibitory Activity and Transcriptional Responses to Nitrate Fluctuation

**FIGURE 2:**
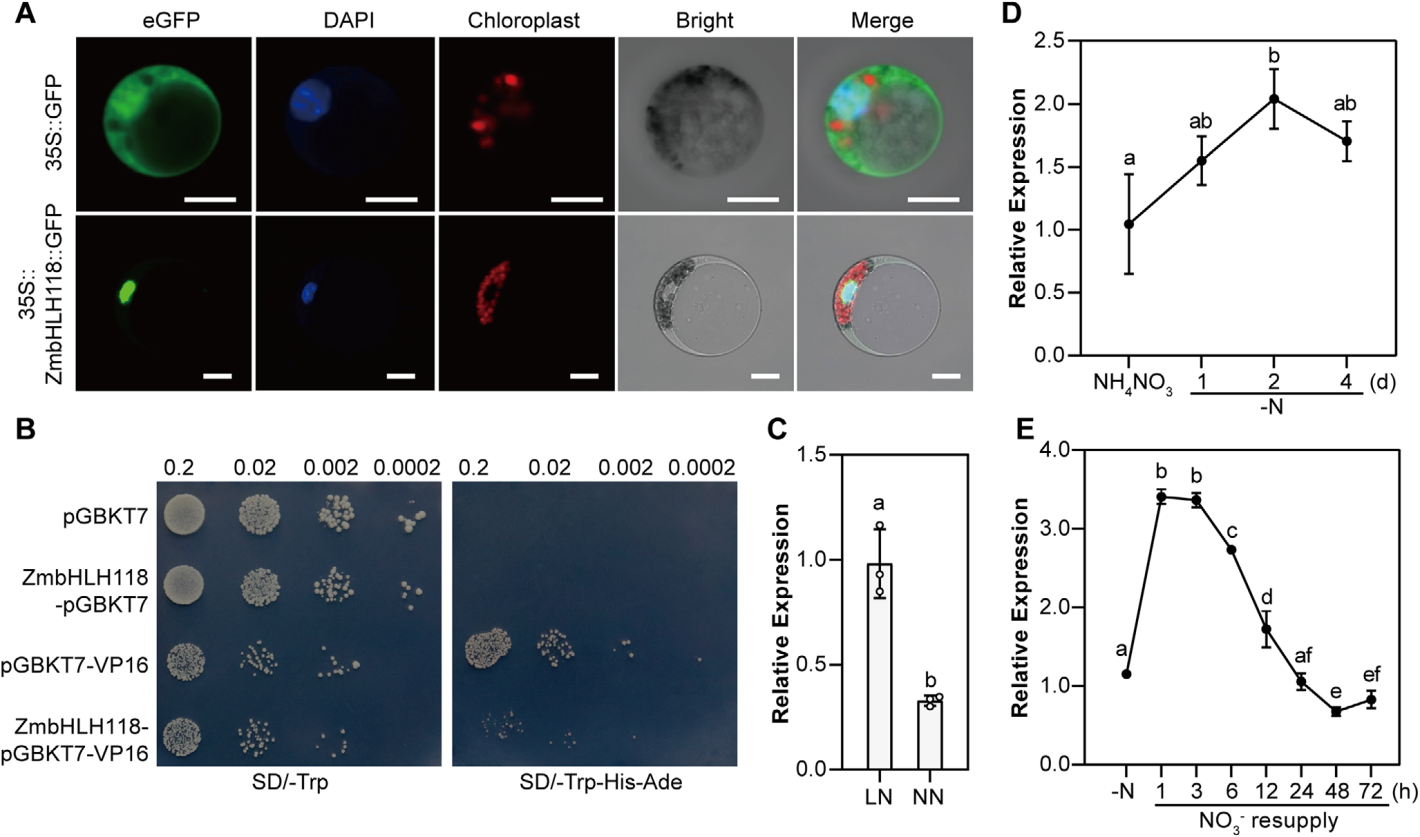
ZmbHLH118 is localized in the nucleus and nitrate negatively regulates its expression. (A) ZmbHLH118 is localized in the nucleus. ZmbHLH118‐GFP or GFP was transiently expressed in maize protoplasts. DAPI (blue signal) was used as a nucleus localization marker. Scale bars, 10 µm. (B) Transcriptional activity analyses of ZmbHLH118. pGBKT7 was used as the negative control, and pGBKT7‐VP16 was used as the positive control. (C) The expression levels of *ZmbHLH118* in maize seedlings grown in LN and NN conditions for two weeks. (D, E) qRT‐PCR assay analyzes the transcript levels of *ZmbHLH118* in the root in response to nitrogen. (D) Wild‐type maize seedlings treated with N‐free (‐N) nutrient solution for 1, 2, or 4 days after 10 days under normal nitrogen (2 mm NH_4_NO_3_) conditions. (E) Four‐day N starvation (‐N) treated wild‐type maize seedlings were resupplied with 4 mm KNO_3_ for 1, 3, 6, 12, 24, 48, or 72 h. *ZmTUB* was used as the internal reference, and the transcript levels of untreated samples were set to 1. Data in (C–E) are means ± SD (*n* = 3 technical replicates). Statistical significance was determined using a two‐tailed Student's *t*‐test in (C) and one‐way ANOVA followed by Tukey's multiple comparison test in (D and E). Different letters represent a significant difference at *p* < 0.05.

ZmbHLH118 belongs to the bHLH family, which has 208 group members in maize plants (Figure S3A). To further explore the characterizations of ZmbHLH118 as a transcription factor, we transiently overexpressed ZmbHLH118 fused with green fluorescent protein (GFP) at the C‐terminus in maize protoplasts driven by the 35S promoter, and found that ZmbHLH118 is localized in the nucleus (Figure 2A). Additionally, we employed the yeast strain Y2HGold (*Saccharomyces cerevisiae*) to determine the transcriptional activity of ZmbHLH118 (Figure 2B). The results showed that, when ZmbHLH118 was expressed, an inhibition of yeast cell growth was observed in the selection medium of SD/‐Trp‐His‐Ade (Figure 2B), indicating that ZmbHLH118 has an inhibitory activity as a transcriptional repressor. Furthermore, we performed a qRT‐PCR assay to determine the organ‐specific expression pattern of *ZmbHLH118* and found that *ZmbHLH118* was predominantly expressed in the root during the seedling stage (Figure S4A) and highly expressed in the leaf during the jointing stage and beyond (Figure S4B–D). The expression pattern shown in the MaizeGDB database indicated that *ZmbHLH118* is predominantly expressed in the roots, both stele and cortex cells (Figure S4E).

Subsequently, to determine if *ZmbHLH118* expression in maize seedlings was influenced by long‐term LN and NN conditions, as well as by N deprivation and nitrate resupply, we analyzed the transcriptional levels of *ZmbHLH118* under different nitrate/nitrogen treatments using qRT‐PCR. The results showed that *ZmbHLH118* was upregulated by the absence of nitrate in the growing media for 2 weeks (Figure 2C). Furthermore, in plants grown for 10 days under 2 mm NH_4_NO_3_, a 2‐day nitrogen deprivation significantly enhanced *ZmbHLH118* expression (Figure 2D). The resupply of 4 mm KNO_3_ after 4‐day nitrogen starvation transiently induced *ZmbHLH118* expression, which indicated that *ZmbHLH118* is nitrate inducible (Figure 2E).

Taken together, these results indicate that ZmbHLH118 functions as a transcription factor with inhibition activity, and that nitrate negatively regulates its expression.

### 2.3 ZmbHLH118 Overexpression Causes Transcriptional Response to Nitrate in Maize

The data above showed that ZmbHLH118 functions as a transcription factor in regulating maize plant growth and nitrate/nitrogen contents. To further investigate the potential target genes of ZmbHLH118 that are involved in the nitrate/nitrogen response, we conducted RNA‐seq analysis on the roots of 3‐week‐old ZmbHLH118‐OE1 and wild‐type maize seedlings grown under LN and NN conditions. Principal component analysis (PCA) (Figure S5A) and correlation heat map analysis (Figure S5B) of three biological replicates indicated that the repeatability among the biological replicates was consistent.

**FIGURE 3:**
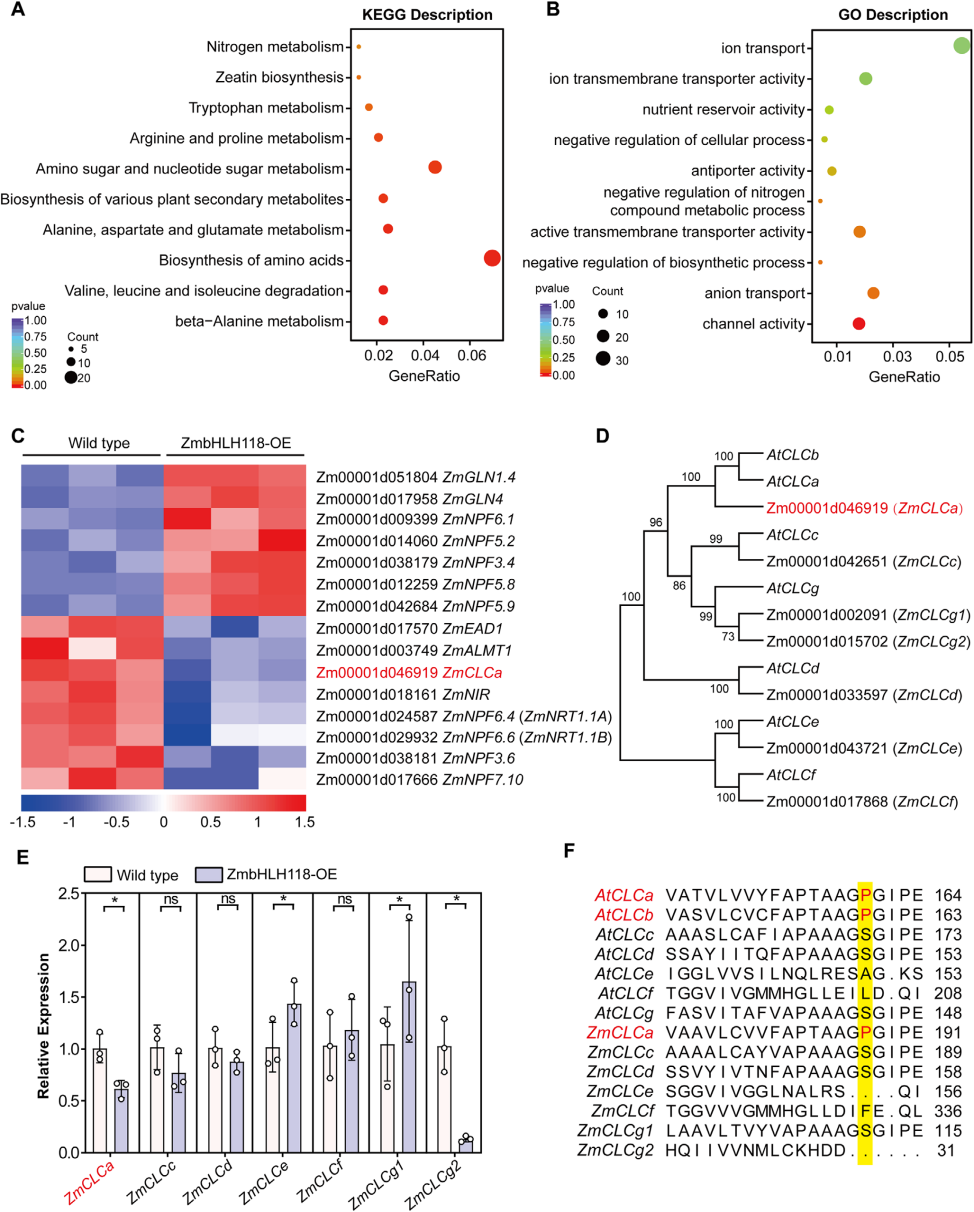
The root transcriptome response to nitrate in ZmbHLH118 overexpressing and wild‐type maize plants. Transcriptomics on roots of 3‐week‐old ZmbHLH118‐OE and wild‐type maize plants treated with Low Nitrate (LN, 0.04 mm KNO_3_) or Normal Nitrate (NN, 4 mm KNO_3_). Differentially expressed genes (DEGs) were filtered for significance (FDR < 0.005). (A) KEGG analysis and (B) GO analysis showed the functional pathway of ZmbHLH118 according to the differentially expressed genes (DEGs) under NN exclude LN. (C) Heat map analysis of nitrogen metabolism and anion transport pathway. (D) Phylogenetic trees of CLC family genes in maize (*n* = 7) and *Arabidopsis* (*n* = 7). A phylogenetic tree was generated using MEGA11. (E) qRT‐PCR assay analyzes the transcript levels of maize CLC family genes in the root of 3‐week‐old wild‐type and ZmbHLH118‐OE plants under NN conditions. *ZmTUB* was used as the internal reference, and the transcript levels of wild type were set to 1. (F) Alignment of the Proline 160 amino acid residue of *Arabidopsis* and maize CLC family genes, which is essential for the substrate selection of nitrate anions [38]. Data in (E) are means ± SD (*n* = 3 biological replicates). Statistical significance was determined using a two‐tailed Student's *t*‐test. ns represents no significant difference (*p* > 0.05), * represents *p* < 0.05.

Then, differentially expressed genes (DEGs) were identified based on the criteria of a significant difference (FDR < 0.005). By comparing the RNA‐seq results between ZmbHLH118‐OE1 and the wild‐type plants, 179 and 2413 DEGs were identified under LN and NN conditions, respectively (Figure S5C). Given the absence of difference in growth and nitrate/nitrogen content between ZmbHLH118 overexpressing and the wild‐type maize plants under LN conditions (Figure 1), we excluded DEGs of LN from NN by overlapping DEGs (Figure S5C). A total of 2322 DEGs were identified, among which 944 were downregulated, and 1378 were upregulated (Figure S5D). Furthermore, to explore the biological processes associated with ZmbHLH118, we performed a Kyoto Encyclopedia of Genes and Genomes (KEGG) and a Gene Ontology (GO) analysis. KEGG analysis revealed significant enrichment of pathways related to nitrogen metabolism, amino acid metabolism, and amino acid biosynthesis. GO analysis highlighted enrichment in processes of ion transport, anion transport, channel activity, and regulation of nitrogen (Figure 3A,B). Based on these results, we assumed ZmbHLH118 might regulate nitrate/nitrogen content in maize by participating in nitrogen metabolism and NO_3_^−^ anion transport. To test this hypothesis, we conducted hierarchical cluster analysis covering the DEGs of nitrogen metabolism and anion transport, revealing a cluster of DEGs including *ZmGLNs*, *ZmNIR*, *ZmEADs*, *ZmNPFs (NRT/PTRs)*, *ZmALMTs*, and *ZmCLCs* (Figure 3C).

Several anion channels/transporters have been reported to be involved in nitrate uptake and homeostasis [26, 29, 54, 81, 82, 83], among which *AtCLCa* has been reported to be localized in the tonoplast and mediates nitrate transport into the vacuole in *Arabidopsis* [32, 33, 41, 42, 84]. Interestingly, one maize CLC family member (Zm00001d046919) was identified by our RNA‐seq analysis (Figure 3C). Therefore, we carried out a phylogenetic analysis by using protein sequences of CLC family members in *Arabidopsis* and Maize, and found that Zm00001d046919 was the closest homolog of the *Arabidopsis AtCLCa* and *AtCLCb* (Figure 3D). We named this maize CLC gene *ZmCLCa* (Zm00001d046919). Next, we conducted qRT‐PCR assays to quantify the gene expression levels of all maize CLCs in ZmbHLH118‐OE1 and wild‐type plants under NN conditions, as we did in the RNA‐seq assay. The experimental results showed that compared to the wild type, the transcript levels of *ZmCLCa* and *ZmCLCg2* were downregulated in the ZmbHLH118‐OE1 plants, while *ZmCLCe* and *ZmCLCg1* were significantly upregulated (Figure 3E), confirming the findings of the downregulation of *ZmCLCa* in our transcriptomics analysis (Figure 3C,E). Notably, Proline 160 in *Arabidopsis* has been identified as the key residue of *AtCLCa* for nitrate selectivity and transport [38]. Thus, we aligned protein sequences of all family members in *Arabidopsis* and Maize, and surprisingly found that only the maize *ZmCLCa* harbors a Proline at the position corresponding to that of *AtCLCa* and *AtCLCb* in *Arabidopsis*, whereas other maize CLCs contain Serine or other residues (Figure 3F). This suggests a potential nitrate transport function for ZmCLCa. Further analyses revealed that *CLCa* homologs in other crop plants (i.e., wheat, rice, and soybean) also possess a Proline at the corresponding position (Figure S6), suggesting that this Proline residue is evolutionarily conserved across diverse plant species.

Previous studies have shown that nitrate uptake across the plasma membrane and cellular nitrate assimilation affect plant nitrate and nitrogen content [61, 83]. Therefore, we performed qRT‐PCR analysis and found that *ZmNRT2.1*, *ZmNR1.1*, and *ZmNIR1.1* were significantly downregulated in ZmbHLH118 overexpressing maize plants compared to the wild type (Figure S7). Taken together, these results indicate that ZmbHLH118 is involved in nitrate metabolism and transport, and that *ZmCLCa* may serve as a transcriptional target of ZmbHLH118.

### 2.4 ZmbHLH118 Directly Binds to the Promoter and Negatively Regulates the Expression of *ZmCLCa*

**FIGURE 4:**
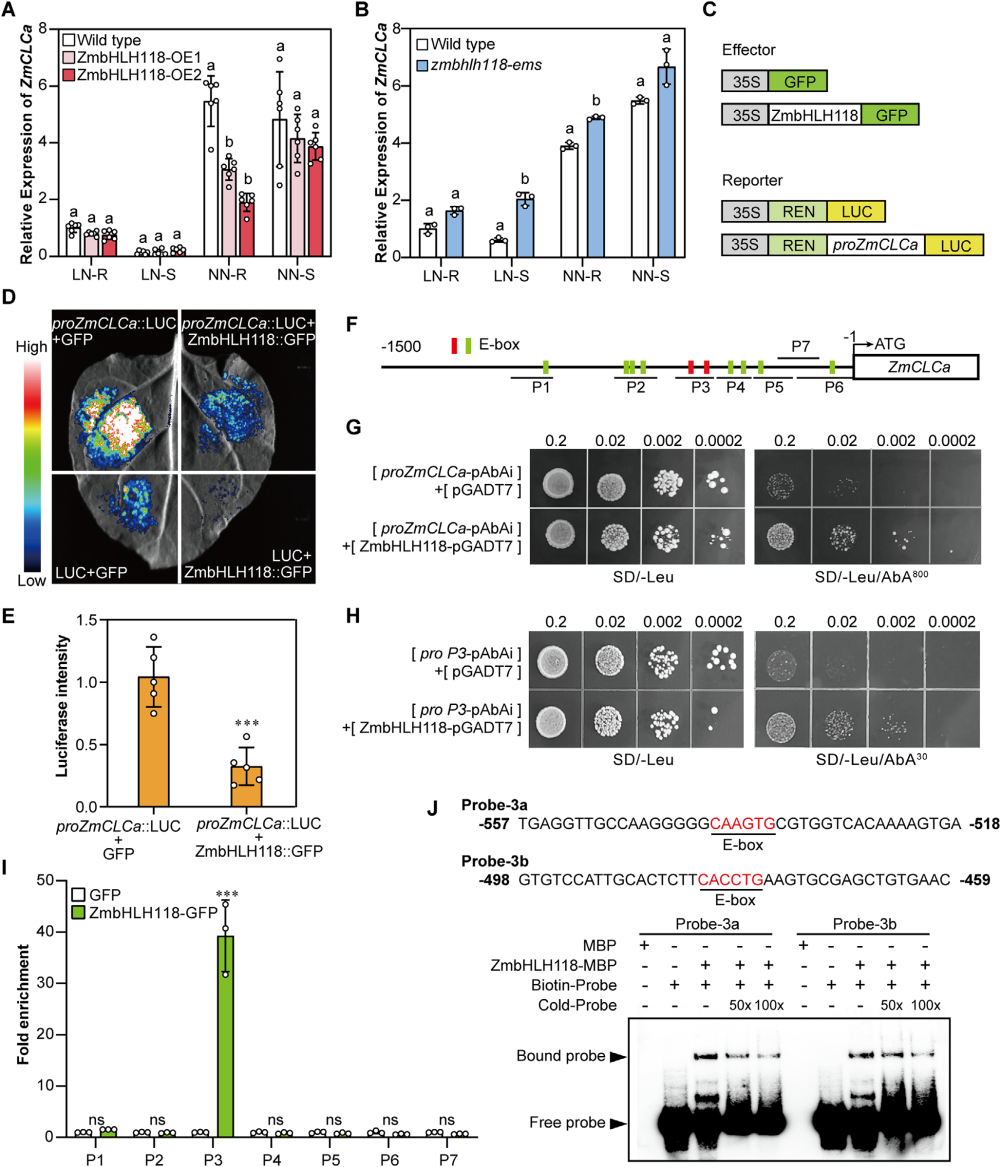
ZmbHLH118 directly binds to the promoter of *ZmCLCa* and negatively regulates its expression. Three‐week‐old wild‐type, ZmbHLH118 overexpressing (A), and *zmbhlh118‐ems* mutant (B) plants were grown under Low Nitrate (LN, 0.04 mm KNO_3_) and Normal Nitrate (NN, 4 mm KNO_3_) conditions, and the relative transcript abundance of *ZmCLCa* in shoot (S) and root (R) tissue was determined by qRT‐PCR analysis. *ZmTUB* was used as the internal reference. Data in (A and B) are means ± SD (*n* = 3–6 technical replicates). Statistical significance was determined using one‐way ANOVA followed by Tukey's multiple comparison test in (A) and two‐tailed Student's *t*‐test in (B). Different letters represent a significant difference at *p* < 0.05. (C) Schematic diagram of the reporter and effector constructs used in the dual luciferase assay. (D) The appearance of dual luciferase assay and (E) the relative luciferase intensity of the *ZmCLCa* promoter co‐expressing with ZmbHLH118 in *Nicotiana benthamiana*. The luciferase intensity was calculated by ImageJ of five biological replicates. The luciferase intensity of *proZmCLCa*::LUC + GFP was set to 1. Data in (E) are means ± SD (*n* = 5 biological replicates). Statistical significance was determined using a two‐tailed Student's *t*‐test. ns represents no significant difference (*p* > 0.05), *** represents *p* < 0.001. (F) The E‐box motifs (colored in red or green) and seven fragments of the promoter of *ZmCLCa* are shown. The fragment that did not contain the E‐box was selected as the negative control and named P7, used in (I). (G, H) Yeast‐one‐hybrid assay for binding of ZmbHLH118 to the promoter of *ZmCLCa*. (G) Full‐length and (H) P3 fragment of the promoter of *ZmCLCa* were examined, and the Aureobasidin A (AbA) was used to inhibit leaky reporter expression at indicated concentrations. (I) Fold enrichment of fragmented DNA of the *ZmCLCa* promoter pulled down by ZmbHLH118‐GFP in the ChIP‐qPCR assay. ZmbHLH118‐GFP was immunoprecipitated from the wild‐type maize protoplasts transiently overexpressing ZmbHLH118‐GFP. GFP was used as a negative control, and its fold enrichment was set to 1. (J) EMSA for examining the binding of ZmbHLH118 to the P3 promoter fragment of *ZmCLCa*. Two E‐box motifs in the P3 promoter fragment of *ZmCLCa* were labeled with biotin and named Probe‐3a and Probe‐3b, respectively. ZmbHLH118‐MBP and MBP (negative control) were incubated with biotin‐labeled probe (Probe‐3a and Probe‐3b) and unlabeled probes used as the competitive probes (Cold‐Probe). The upper arrow indicates biotin‐labeled probes bound to ZmbHLH118‐MBP. Data in (I) are means ± SD (*n* = 3 technical replicates). Statistical significance was determined using a two‐tailed Student's *t*‐test. ns represents no significant difference (*p* > 0.05), *** represents *p* < 0.001.

The RNA‐seq results showed that *ZmCLCa* is a potential transcriptional target of ZmbHLH118. To validate the regulation of ZmbHLH118 on *ZmCLCa*, we employed ZmbHLH118 transgenic plants, including ZmbHLH118‐OE1, ZmbHLH118‐OE2, and *zmbhlh118‐ems*, to analyze the transcript levels of *ZmCLCa* by qRT‐PCR under LN and NN conditions. After 2‐week growth in hydroponic culture, no difference in *ZmCLCa* expression was detected in either roots or shoots between ZmbHLH118‐OE and wild‐type plants under LN conditions (Figure 4A). However, under NN conditions, *ZmCLCa* exhibited significantly lower transcript levels in the roots of ZmbHLH118‐OE1 and ZmbHLH118‐OE2 plants than those in the wild type, but not in the shoots (Figure 4A). Next, we examined *ZmCLCa* expression in the *zmbhlh118‐ems* mutant and found that the expression of *ZmCLCa* was higher in the *zmbhlh118‐ems* mutant under both LN and NN conditions, with significance in the shoots under LN and the roots under NN treatment (Figure 4B).

To further explore whether the ZmbHLH118 protein directly binds to the promoter of *ZmCLCa*, we conducted a dual luciferase assay in *Nicotiana benthamiana* by using a 1.5‐kb promoter fragment of *ZmCLCa* (Figure 4C). When co‐infiltrating the leaves with *Agrobacterium tumefaciens* harboring the ZmbHLH118‐GFP effector construct and the *proZmCLCa*::LUC reporter construct, a significant decrease in luciferase activity derived from *proZmCLCa*::LUC was observed (Figure 4D), and the relative luciferase intensity was reduced by 69% (Figure 4E), indicating that ZmbHLH118 inhibits *ZmCLCa* expression via directly binding to its promoter.

bHLH transcription factors preferentially bind to the motifs of E‐box (5′‐CANNTG‐3′) elements to regulate the expression of the target genes [79]. Using PlantPAN 4.0, we performed motif analysis of the *ZmCLCa* promoter and identified ten conserved E‐box motifs. Based on this analysis, the promoter was divided into seven fragments (P1‐P7) (Figure 4F) for further analysis. P1‐P6 contained one to three E‐box motifs, whereas P7, which lacks an E‐box motif, was selected as the negative control (Figure 4F).

To investigate whether ZmbHLH118 directly binds to the identified E‐box motifs of the *ZmCLCa* promoter, we carried out a yeast‐one‐hybrid (Y1H) assay. The Y1HGold (*Saccharomyces cerevisiae*) co‐transformed with the *proZmCLCa*‐pAbAi and ZmbHLH118‐pGADT7 constructs grew significantly better than the cells harboring *proZmCLCa*‐pAbAi constructs on the SD/‐Leu medium with the application of 800 ng/mL Aureobasidin A (AbA) (Figure 4G). These results confirmed that ZmbHLH118 directly binds to *ZmCLCa* promoter. Furthermore, similar Y1H assays were conducted with the co‐transformed P1‐P6 fragments of *ZmCLCa* promoter with ZmbHLH118‐pGADT7 and revealed that only the cells containing the P3 fragment grew better than the control, as the full‐length promoter of *ZmCLCa* did (Figure 4H; Figure S8), indicating that ZmbHLH118 potentially binds to the P3 fragment of *ZmCLCa* promoter. Next, we performed chromatin immunoprecipitation (ChIP) assays by transiently overexpressing ZmbHLH118‐GFP in maize protoplasts. Immunoprecipitation was carried out using an anti‐GFP antibody, followed by qRT‐PCR to assess the enrichment of the P1‐P6 DNA fragments. Using P7 as the negative control, the results showed that ZmbHLH118‐GFP specifically bound to the P3 fragment, with a 39‐fold enrichment (Figure 4I), confirming that ZmbHLH118 binds to the P3 fragment of the *ZmCLCa* promoter.

P3 fragment contains two E‐box motifs (Figure 4F). Then, we performed electrophoretic mobility shift assays (EMSAs) to explore which of the two E‐box motifs in the P3 fragment is the binding site for ZmbHLH118 (Figure 4F). We synthesized a 40‐bp oligonucleotide and labeled it with biotin as probes, named Probe‐3a and Probe‐3b, respectively (Figure 4J). The unlabeled probes were used as competitors (Cold‐Probe). After co‐incubating ZmbHLH118‐MBP protein with the labeled probe, direct binding of ZmbHLH118‐MBP on Probe‐3a and Probe‐3b was observed (Figure 4J). Moreover, the reduction of ZmbHLH118 binding to the labeled probe was clearly detected with the increasing application of unlabeled probes (Figure 4J).

In maize, ZmbHLH118 shares similar amino acid sequences with the other three bHLH transcription factors, ZmbHLH162 (GRMZM2G093744), ZmbHLH164 (GRMZM2G058451), and ZmbHLH172 (GRMZM2G017586) (Figure S3). To determine whether these three bHLHs also negatively regulate *ZmCLCa* expression, we conducted a dual luciferase assay in *Nicotiana benthamiana* by co‐infiltrating the leaves with *Agrobacterium tumefaciens* harboring the ZmbHLH::GFP effector construct and the *proZmCLCa*::LUC reporter construct. We found that ZmbHLH162, ZmbHLH164, and ZmbHLH172 significantly attenuated the luciferase intensity derived from *proZmCLCa*::LUC, similarly to ZmbHLH118 (Figure S9), suggesting the possible existence of functional redundancy among these ZmbHLHs.

Taken together, our data indicated that ZmbHLH118 directly binds to the promoter of *ZmCLCa* and negatively regulates its transcript levels.

### 2.5 ZmCLCa is Localized in the Tonoplast and Transcriptionally Responds to Nitrate

**FIGURE 5:**
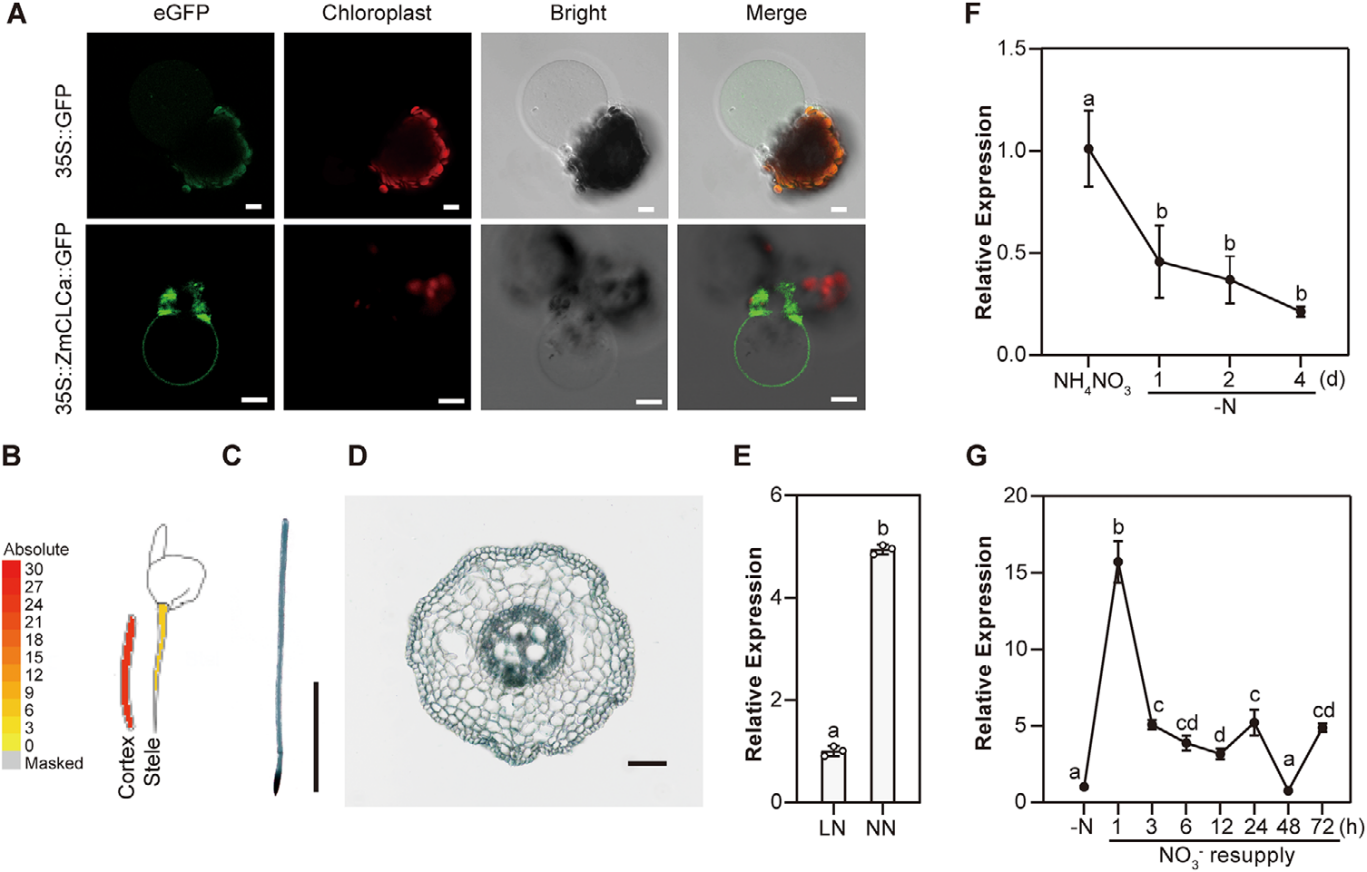
ZmCLCa is localized in the tonoplast and nitrate positively regulates its expression. (A) Subcellular localization of ZmCLCa in the tonoplast of maize protoplast with the released vacuole. The vacuole was released using the solution (0.1 mm CaCl_2_, 3 mm MgCl_2_, 100 mm HCl, moderate BisTrisPropane to pH 7.5, 500 mOsm). Scale bars, 10 µm. (B) The heat map shows the transcript levels of *ZmCLCa* in the root cortex and stele tissues. The figure was modified from (https://www.maizegdb.org). (C) Expression pattern of a *promoter*::GUS reporter construct of *ZmCLCa* and (D) transsection of stained root. The root of a two‐leaf‐old *proZmCLCa*‐GUS maize seedling was incubated in GUS solution after vacuum treatment. Scale bars, 1 cm in (C) and 10 µm in (D). (E) The expression levels of *ZmCLCa* in maize seedlings grown in LN and NN conditions for two weeks. (F, G) qRT‐PCR assay analyzes the transcript levels of *ZmCLCa* in the root in response to nitrogen. (F) Wild‐type maize seedlings treated with N‐free (‐N) nutrient solution for 1, 2, or 4 days after 10 days under normal nitrogen (2 mm NH_4_NO_3_) conditions. (G) Four‐day N starvation (‐N) treated wild‐type maize seedlings were resupplied with 4 mm KNO_3_ for 1, 3, 6, 12, 24, 48, or 72 h. *ZmTUB* was used as the internal reference, and the transcript levels of untreated samples were set to 1. Data in (E‐G) are means ± SD (*n* = 3 technical replicates). Statistical significance was determined using two‐tailed Student's *t*‐test in (E) and one‐way ANOVA followed by Tukey's multiple comparison test in (F and G). Different letters represent a significant difference at *p* < 0.05.

CLCa and CLCb have been reported to localize on the vacuolar membrane in *Arabidopsis* [33, 42]. Transient expression of C‐terminal GFP‐tagged ZmCLCa (35S::ZmCLCa::GFP) in maize protoplasts showed that it localizes in the tonoplast (Figure 5A).

To investigate the organ‐specific expression pattern of *ZmCLCa*, qRT‐PCR analysis was conducted, showing comparable expression levels of *ZmCLCa* in roots and shoots at the seedling stage (Figure S10A). At the jointing stage and during subsequent developmental stages, *ZmCLCa* shows high expression in the leaf (Figure S10B–D). The expression pattern shown in the MaizeGDB database indicated that *ZmCLCa* is predominantly expressed in root cortex and stele cells (Figure 5B). To determine the tissue‐specific promoter activity of *ZmCLCa*, we fused a 3‐kb promoter fragment of *ZmCLCa* with the *GUS* reporter gene (*proZmCLCa*::GUS). GUS staining showed that the *ZmCLCa* was mainly expressed in roots (Figure 5C), and transect slices showed that *ZmCLCa* was expressed throughout the roots, including the epidermis, the cortex, and the stele (Figure 5D).

Previous reports have shown that application of nitrate triggers *AtCLCa* expression in *Arabidopsis* [32]. We therefore tested if *ZmCLCa* expression in maize seedlings was also modified by long‐term LN and NN conditions, as well as in response to N deprivation and nitrate resupply. qRT‐PCR assays showed that *ZmCLCa* was upregulated by the presence of nitrate in the growing media (Figure 5E). In contrast, N deprivation for 1, 2, or 4 days resulted in significant downregulation of *ZmCLCa* transcript levels (Figure 5F), while NO_3_^−^ resupply rapidly induced its expression as early as 1 h after 4 days of N starvation (Figure 5G). These results indicated that nitrate positively regulates *ZmCLCa* transcript levels in maize.

### 2.6 ZmCLCa Positively Regulates the Maize Growth and Nitrate Content

**FIGURE 6:**
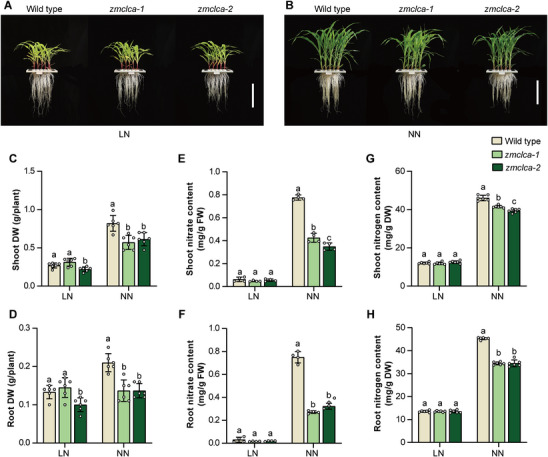
ZmCLCa positively regulates maize growth and nitrate content. Three‐week‐old *zmclca* knockout mutants (*zmclca‐1*, *zmclca‐2*) and wild‐type maize plants growth under (A) Low Nitrate (LN, 0.04 mm KNO_3_) and (B) Normal Nitrate (NN, 4 mm KNO_3_) conditions. (C, D) The biomass, (E, F) nitrate concentration, and (G, H) nitrogen concentration in shoot and root tissue of *zmclca* knockout mutants and wild‐type maize plants grown in the indicated nitrate conditions. DW, dry weight; FW, fresh weight. (A and B) Scale bars, 20 cm. Data in (C–H) are means ± SD (*n* = 4–6 biological replicates). Statistical significance was determined using one‐way ANOVA followed by Tukey's multiple comparison test. Different letters represent a significant difference at *p* < 0.05.

Next, to further explore the functions of ZmCLCa in maize, we generated two independent loss‐of‐function mutants using CRISPR/Cas9, designated *zmclca‐1* and *zmclca‐2*. The *zmclca‐1* mutant harbors a single‐nucleotide (A) insertion that causes a frameshift, whereas *zmclca‐2* harbors a two‐nucleotide deletion resulting in a truncated ZmCLCa protein (Figure S11). Phenotypic analysis of 3‐week‐old *zmclca‐1* and *zmclca‐2* plants under LN and NN conditions showed that growth was significantly impaired under NN conditions, but not under LN conditions (Figure 6A,B). *zmclca‐1* and *zmclca‐2* plants exhibited significantly reduced root and shoot biomass compared to wild‐type plants under NN conditions (Figure 6C,D). Remarkably, unlike the LN conditions, *zmclca‐1* and *zmclca‐2* mutants grown under NN conditions displayed a significantly reduced nitrate content than the wild type, with a 45% and 55% decrease in the shoots and a 64% and 57% decrease in the roots, respectively (Figure 6E,F). A significant reduction of nitrogen content in the shoots and roots of *zmclca‐1* and *zmclca‐2* plants was also detected (Figure 6G,H). Compared with the shoots, roots of *zmclca* mutants showed a more pronounced reduction in nitrate and nitrogen contents (Figure 6E–H), consistent with observations in ZmbHLH118 overexpressing plants (Figure 1E–H).

Additionally, we obtained an EMS‐induced mutagenesis mutant of ZmCLCa*, zmclca‐ems*, which harbors a G substitution to A at the position of 691st amino acid residue in the third exon, resulting in a truncated ZmCLCa protein (Figure S12A). The *zmclca‐ems* mutant also displayed a growth impairment under NN conditions (Figure S12B,C), showing comparable results to *zmclca‐1* and *zmclca‐2* mutants with significant biomass, nitrate, and nitrogen content reductions (Figure S12D–I).

Overexpression of ZmCLCa in maize, in which the transcript levels of *ZmCLCa* were 128‐fold (ZmCLCa‐OE1) and 145‐fold (ZmCLCa‐OE2) increased (Figure S13A), resulted in plants with growth comparable to wild‐type plants under both LN and NN conditions (Figure S13B,C). Only ZmCLCa‐OE2 plants showed a slight biomass increase of 28% in the shoots and 42% in roots (Figure S13D,E). However, no significant differences in nitrate and nitrogen content were detected between either ZmCLCa overexpressing line and wild‐type plants (Figure S13F–I).

In summary, these findings suggested that ZmCLCa promotes maize growth and nitrate/nitrogen content in NN conditions when NO_3_^−^ is highly available.

### 2.7 ZmCLCa Promotes Nitrate Uptake in Maize Roots and Chlorate Resistance

**FIGURE 7:**
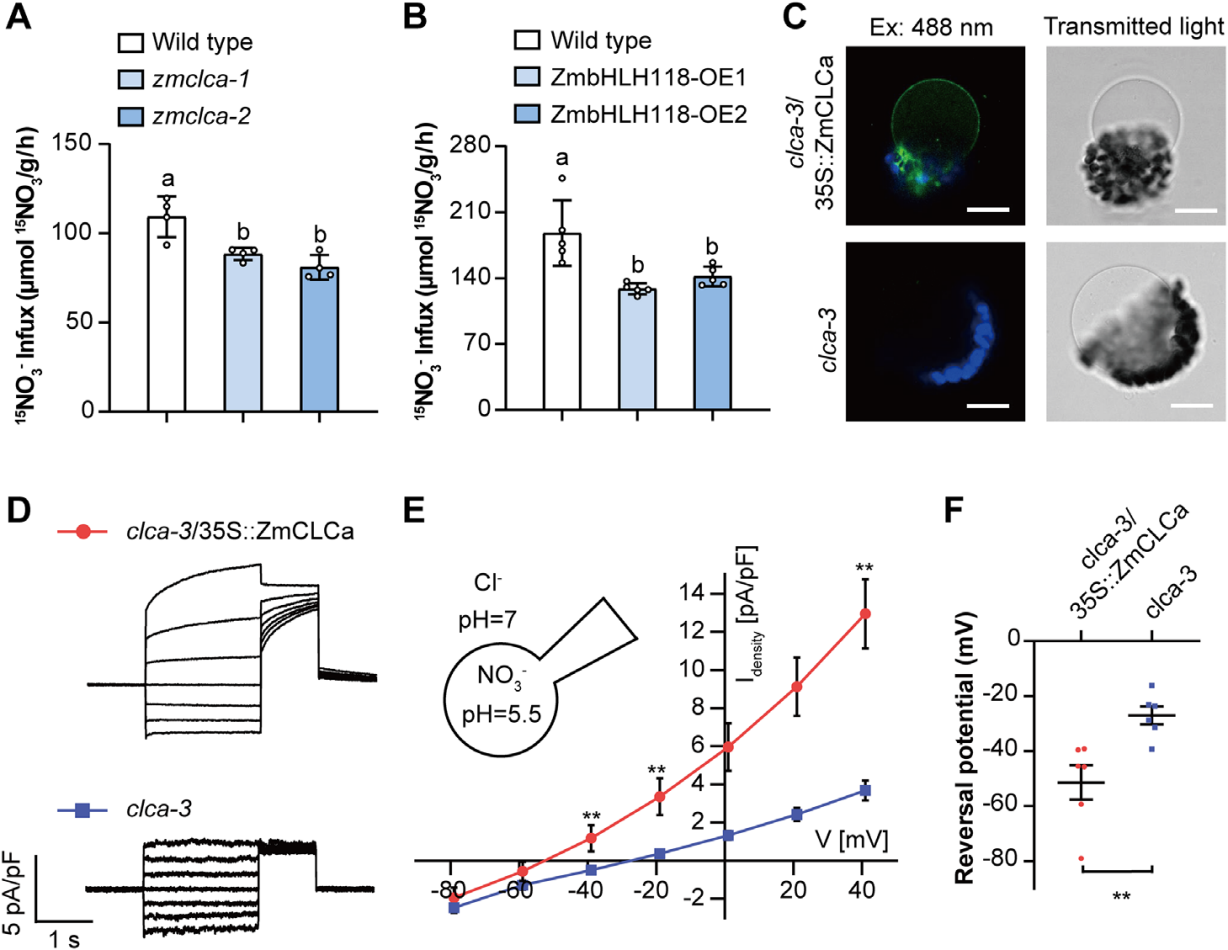
ZmCLCa promotes nitrate uptake in maize roots and restores nitrate currents in *Arabidopsis clca‐3* mutant. (A) *zmclca* knockout mutants and (B) ZmbHLH118 overexpressing plants showed ZmCLCa and ZmbHLH118 played the opposite roles in nitrate uptake. Maize seedlings were cultured with 4 mm KNO_3_ for 10 days and then transferred to N‐free nutrient solution for 4 days, and resupplied 4 mm KNO_3_ for 3 h, finally cultured with 4 mm 10% ^15^N‐labelled KNO_3_ for 6 min. Roots were prepared for detection. (C) Confocal images of vacuoles from transiently transfected protoplasts with ZmCLCa with GFP tag (upper panel) or untransfected (lower panel). GFP: green; Chlorophyll: blue. Scale bars, 20 µm. (D) Representative currents from whole vacuole patch clamp recordings overexpressing ZmCLCa in the *Arabidopsis* knocked out *clca‐3* (upper panel) and *clca‐3* (lower panel). Voltage pulses were applied for 2 s from −79 to +41 mV in +20 mV increments; a post pulse of −69 mV was applied after each stimulation for 1 s. The holding potential was −19 mV. (E) Current–voltage curves of the mean current densities in whole vacuole patches overexpressing ZmCLCa and in *clca‐3*. (F) Reversal potentials from vacuoles overexpressing ZmCLCa and *clca‐3*. Cytosolic pH 7 with 19.2 mm Cl^−^, vacuolar pH 5.5 with 200 mm NO_3_^−^. Data in (E and F) are means ± SEM (*n* = 6 vacuoles). Statistical significance was determined using the Mann–Whitney nonparametric test. ** represents a significant difference at *p* < 0.01.

Given the findings that ZmCLCa is localized on the vacuolar membrane (Figure 5A) and remarkably reduced nitrate content in *zmclca* mutant roots (Figure 6E–H; Figure S12F–I), we hypothesized that interruption of vacuole nitrate loading in the roots and shoots can potentially impair the root NO_3_^−^ uptake. To address this hypothesis, we carried out a ^15^N‐labelled NO_3_^−^ influx assay employing the *zmclca* knock‐out mutants (*zmclca‐1* and *zmclca‐2*). After 4 days of nitrate starvation on the maize seedlings under NN conditions for 10 days, 4 mm NO_3_^−^ was fed for 3 h, and then 4 mm ^15^N‐labelled NO_3_^−^ was applied for 6 min. The measurements of ^15^N in roots showed a significantly decreased ^15^N‐labelled NO_3_^−^ influx rate in *zmclca‐1* and *zmclca‐2* mutants compared to the wild‐type plants (Figure 7A). Then, we performed the same assay using ZmbHLH118 overexpressing maize plants (ZmbHLH118‐OE1 and ZmbHLH118‐OE2) and found that ZmbHLH118‐OE1 and ZmbHLH118‐OE2 plants displayed a significantly lower ^15^N‐labelled NO_3_^−^ influx rate in roots than the wild‐type plants (Figure 7B), similar to the *zmclca* mutant plants (Figure 7A). These results indicated that functional interruption of ZmCLCa or transcriptional inhibition of *ZmCLCa* by ZmbHLH118 impairs NO_3_^−^ uptake in the maize root.

Chlorate (ClO_3_^−^) resistance assay has been employed to characterize nitrate transport activity since the absorption and assimilation pathways of chlorate are the same as those of nitrate. Chlorate is reduced by nitrate reductase to toxic chlorite (ClO_2_^−^) rather than innocuous nitrite (NO_2_^−^), resulting in growth inhibition in plants [17, 85, 86, 87]. To obtain further evidence of the role of ZmCLCa in transporting NO_3_^−^, we tested the chlorate resistance of *zmclca* and ZmbHLH118‐OE maize plants (Figure S14). One week after germination, seedlings were cultivated in different chlorate conditions (0 mm and 4 mm KClO_3_). Shoot fresh weight was measured when significant differences were observed between transgenic and wild‐type maize plants. *zmclca* knockout mutants (*zmclca‐1*, *zmclca‐2*), ZmbHLH118 overexpressing (ZmbHLH118‐OE1, ZmbHLH118‐OE2), and wild‐type plants grew healthily with green leaves under chlorate‐free conditions (Figure S14A,C). However, after chlorate treatment, *zmclca* knockout mutants (Figure S14A) and ZmbHLH118 overexpressing seedlings (Figure S14C) exhibited increased susceptibility to chlorate toxicity, showing more severe growth inhibition accompanied by leaf bleaching, withering, and seedling death in some cases. Further measurements showed reduced chlorate resistance in *zmclca* mutants and ZmbHLH118 overexpressing plants, with a 38% and 50% decrease in *zmclca‐1* and *zmclca‐2* (Figure S14B), and 24% and 21% in ZmbHLH118‐OE1 and ZmbHLH118‐OE2 (Figure S14D), respectively. These results indicate that functional interruption of ZmCLCa on the vacuolar membrane impairs maize plant chlorate resistance and may provide insights into the role of ZmCLCa in nitrate transport in plant cells.

### 2.8 ZmCLCa Mediates NO_3_^−^ Transport Across the Vacuolar Membrane

The above results indicate that ZmCLCa most likely functions in mediating nitrate transport into the vacuole, similarly to AtCLCa in *Arabidopsis* [33, 41]. To test this hypothesis, we performed patch‐clamp assays using *Arabidopsis clca‐3 mutant* [24]. In *Arabidopsis clca‐3* protoplasts transiently expressing 35S::ZmCLCa::GFP, the subcellular localization of ZmCLCa‐GFP was in the tonoplast (Figure 7C). Next, we investigated the ion transport capacities of ZmCLCa across the vacuolar membranes by applying the patch‐clamp technique to vacuoles extracted from *clca‐*3 protoplasts expressing 35S::ZmCLCa::GFP in the whole‐vacuole configuration.

Under bi‐ionic conditions (200 mm NO_3_^−^ in the vacuole, 19.2 mm Cl^−^ on the cytosolic side), and in whole‐vacuole configuration, the current density in vacuoles expressing ZmCLCa was 3.5 times higher than in vacuoles from *clca‐3* mutant at +43 mV (Figure 7D). Notably, the ionic currents observed in ZmCLCa expressing vacuoles displayed a time‐dependent kinetics at positive membrane potentials (Figure 7D) similar to the AtCLCa currents [33, 41]. The steady‐state I–V curves showed that ZmCLCa expressing vacuoles displayed a far more negative reversal potential (E_rev_ = −51 ± 15 mV) compared to *clca‐3* vacuoles (E_rev_ = −27 ± 8 mV), in line with the presence of an ion transporter imposing its reversal potential (Figure 7E,F). Overall, the expression of ZmCLCa induced ionic currents similar to those of AtCLCa, suggesting similar ion transport functions [33, 41].

Additionally, we transiently expressed ZmCLCa‐GFP in *Nicotiana benthamiana* where it was also localized in the vacuolar membrane (Figure S15A). We performed patch‐clamp experiments in the whole‐vacuole configuration on tobacco vacuoles as previously described. In these experiments, the vacuoles were exposed to NO_3_^−^ conditions (100 mm NO_3_^−^ in the vacuole, 4.2 mm NO_3_^−^ on the cytosolic side). We could observe a four times higher current density in ZmCLCa overexpressing vacuoles (26.8 ± 1.8 pA/pF at +60 mV) compared to untransformed vacuoles (6.7 ± 0.8 pA/pF at +60 mV) (Figure S15B,C), also similar to the AtCLCa currents [41].

Taken together, these electrophysiological analyses of the ZmCLCa ion transport capacities show that ZmCLCa exhibits biophysical properties that are comparable to AtCLCa and therefore that it is able to mediate nitrate transport into the vacuole.

### 2.9 ZmbHLH118 and ZmCLCa Modulate the Maize Growth and Yield in the Field

**FIGURE 8:**
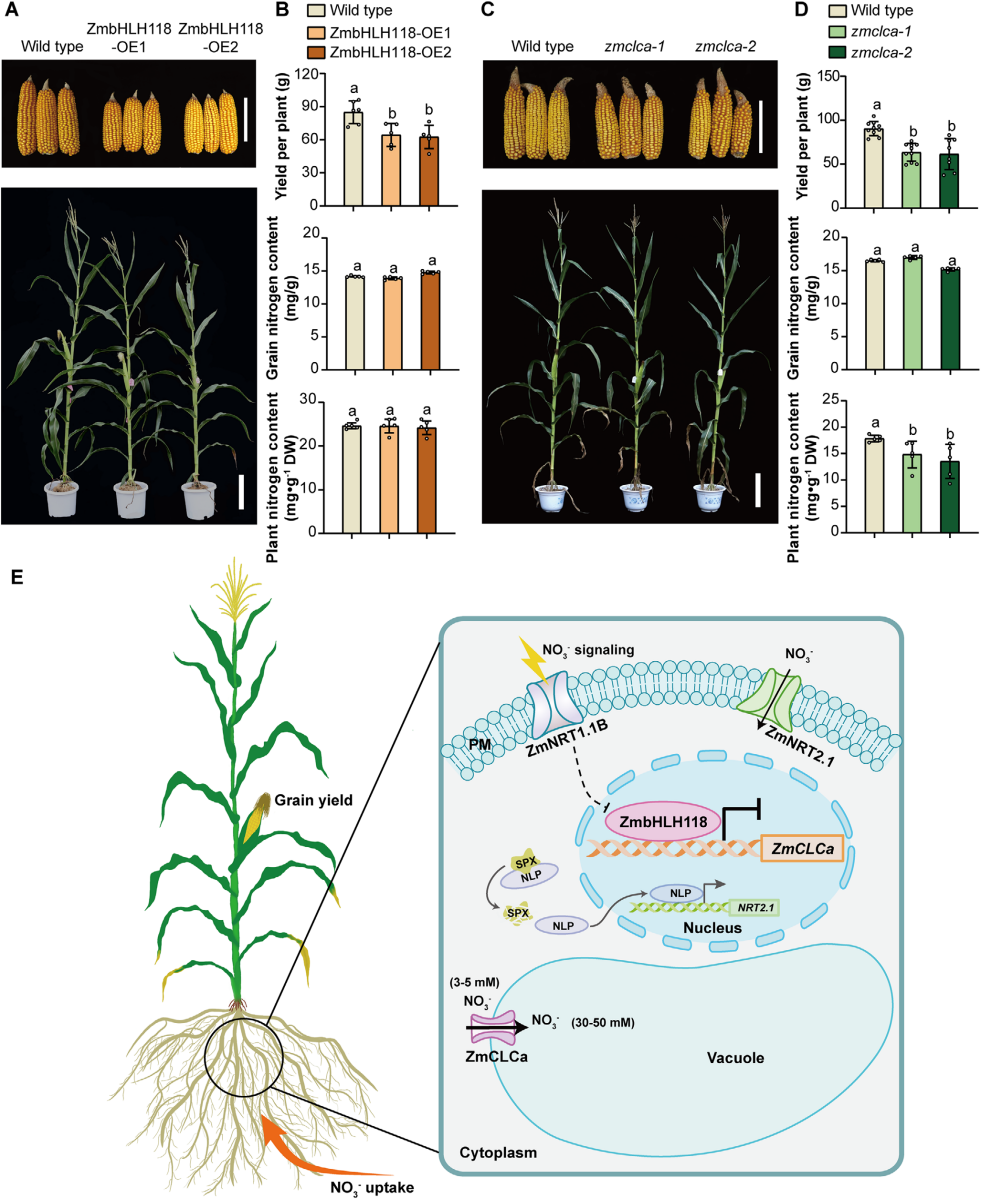
Overexpression of ZmbHLH118 and function loss of ZmCLCa impair maize growth and yield in the field. (A) Representative photographs of ears (Scale bars, 10 cm) at the maturation stage and plants (Scale bars, 30 cm) at the silking stage of ZmbHLH118 overexpressing plants (wild type: ND101). (B) Grain yield per plant, nitrogen content of plants at the silking stage, and nitrogen content of grains at the maturation stage of ZmbHLH118 overexpressing plants in the field (Sanya, Hainan). (C) Representative photographs of ears (Scale bars, 10 cm) at the maturation stage and plants (Scale bars, 30 cm) at the silking stage of *zmclca* knockout mutants (wild type: ND101). (D) Grain yield per plant, nitrogen content of plants at the silking stage, and nitrogen content of grains at the maturation stage of *zmclca* knockout mutants in the field (Shangzhuang, Beijing). Data in (B and D) are means ± SD (*n* ≥ 5 biological replicates). Statistical significance was determined using one‐way ANOVA followed by Tukey's multiple comparison test. Different letters represent a significant difference at *p* < 0.05. (E) Conceptual working model of ZmbHLH118‐ZmCLCa module in maize. In the presence of nitrate, external nitrate is sensed by the plasma membrane‐localized sensor ZmNRT1.1B. The ZmNRT1.1B‐ZmSPXs‐ZmNLP3 module integrates nitrate signaling to activate the plasma membrane‐localized nitrate transporters ZmNRT2.1. Meanwhile, ZmNRT1.1B‐mediated nitrate signaling may repress the expression of *ZmbHLH118* in some way, thereby releasing the inhibition of the tonoplast‐localized nitrate transporter ZmCLCa and enhancing nitrate influx into the vacuole. Nitrate influx into cytoplasm via ZmNRT2.1 in the plasma membrane and efflux from cytoplasm via ZmCLCa in the vacuolar membrane promote the nitrate uptake in the maize roots.

The data above showed pronounced differences in growth, nitrate uptake, and nitrate/nitrogen contents in ZmbHLH118 and ZmCLCa transgenic seedlings (Figures 1 and 6 and 7A,B; Figures S2 and S12 and S13). To understand the impact of ZmbHLH118 and ZmCLCa at the adult stage in the field, we evaluated the yield performance of two independent ZmbHLH118 and ZmCLCa transgenic lines under adequate nutrient conditions in the field. Field trials showed that ZmbHLH118 overexpression significantly suppressed maize growth in the field, as demonstrated by reduced plant height (Figure S16A) and smaller ears (Figure 8A). Further measurements showed that the yields (yield per plant) of ZmbHLH118‐OE1 and ZmbHLH118‐OE2 were reduced by 24% and 26%, respectively, compared with the wild type (Figure 8B). In contrast, *zmbhlh118‐ems* mutant plants were higher (Figure S16B) and yielded with an increase of 18% than the wild type (Figure S17A,B). However, nitrogen contents in plant tissue were not significantly altered in ZmbHLH118 overexpressing (Figure 8B) and *zmbhlh118‐ems* maize plants (Figure S17B). Interestingly, a higher grain nitrogen content was detected in *zmbhlh118‐ems* plants (Figure S17B) rather than ZmbHLH118 overexpressing plants (Figure 8B). Moreover, *zmclca‐1* and *zmclca‐2* mutants exhibited growth inhibition in the field with lower plant height (Figure S16C) and smaller ears (Figure 8C), and the yields of *zmclca‐1* and *zmclca‐2* were reduced by 30% and 32% compared to the wild type, respectively (Figure 8D). A significant reduction of nitrogen contents in plant tissue was observed in *zmclca‐1* and *zmclca‐2* mutant plants, but not in grains (Figure 8D). However, no field growth phenotype and grain/plant tissue nitrogen contents were detected in ZmCLCa overexpressing plants (Figures S16D and S17C,D). These results indicate that ZmbHLH118 and ZmCLCa contribute to field yield and may influence nitrogen use efficiency in maize.

### 2.10 ZmbHLH118 Regulation on ZmCLCa Is Downstream of ZmNRT1.1B‐Mediated NO_3_^−^ Signaling but Independent of ZmNLP3

The plasma membrane localized transceptor NRT1.1 and the intracellular sensor NLP work together to sense and transduce nitrate signaling in plants [51, 52, 53, 61, 62]. To investigate whether NRT1.1‐ or/and NLPs‐mediated nitrate signaling modulates *ZmCLCa* via ZmbHLH118 to regulate intracellular nitrate homeostasis, we employed *zmnrt1.1b* and *zmnlp3.1* mutant plants to examine the transcript levels of *ZmbHLH118* and *ZmCLCa* in response to nitrate. qRT‐PCR analysis showed that high nitrate inhibition on *ZmbHLH118* expression was impaired in *zmnrt1.1b‐1* and *zmnrt1.1b‐2* mutant plants (Figure S18A), while the upregulation of *ZmCLCa* by high nitrate was defective in *zmnrt1.1b* mutants compared to the wild type (Figure S18B). Using qRT‐PCR analysis, we detected a downregulation of *ZmNRT1.1B* in ZmbHLH118 overexpressing maize plants (Figure S18C), like in RNA‐seq assay (Figure 3C), suggesting a possible feedback regulation of ZmbHLH118 on *ZmNRT1.1B*. Differently, qRT‐PCR analysis did not detect a significant difference in the transcript levels of *ZmCLCa* in *zmnlp3.1‐1* and *zmnlp3.1‐2* mutant plants (Figure S19A). Additionally, dual luciferase assay in *Nicotiana benthamiana* showed that ZmNLP3.1 did not affect the luciferase activity driven by *ZmCLCa* promoter (Figure S19B–D). These results indicate that ZmbHLH118‐mediated regulation of *ZmCLCa* functions downstream of ZmNTR1.1‐mediated NO_3_^−^ signaling and independently of ZmNLP3.

## 3 Discussion

Nitrogen is an essential macronutrient and a major limiting factor for plant growth and crop productivity, with nitrate (NO_3_^−^) serving as the dominant nitrogen form taken up from the soil by most terrestrial plants [4, 88]. Vacuoles are the primary intracellular nitrate storage, and transport of NO_3_^−^ across the vacuolar membrane plays a vital role in maintaining cytoplasmic nitrate homeostasis and remobilization, which helps plants to cope with the external NO_3_^−^ availability and metabolic activities [21]. However, tonoplast‐localized nitrate transporters are still poorly characterized in crops, and their upstream regulatory mechanisms remain largely unknown. In this study, we identified a key transcription factor, ZmbHLH118, that modulates nitrate content and uptake in maize plants (Figures 1 and 7B; Figure S2). Moreover, we revealed a ZmbHLH118‐ZmCLCa module for the regulation of vacuolar NO_3_^−^ loading and intracellular nitrate homeostasis (Figure 8E). ZmbHLH118 directly binds to the promoter of *ZmCLCa* and inhibits its expression (Figure 4). *ZmCLCa* encodes a vacuolar nitrate transporter and mediates NO_3_^−^ influx into the vacuole (Figure 7D–F; Figure S15). This study identifies an uncovered mechanism underlying nitrate signaling and controlling vacuolar membrane NO_3_^−^ transport to maintain NO_3_^−^ homeostasis in maize plants.

Previous studies have demonstrated that transcriptional regulation plays a critical role in enabling plants to cope with fluctuations in nitrogen availability [50, 53, 84, 89, 90]. Several critical transcriptional regulators have been identified and characterized, involved in nitrogen uptake and metabolism, root growth and development, carbon metabolism, and hormone pathways [50, 61, 62, 71, 77, 89, 91]. In this study, we discovered that ZmbHLH118 participates in regulating intracellular nitrate homeostasis and nitrate uptake in maize (Figures 1 and 7B; Figure S2). Compared to the wild type, ZmbHLH118 overexpressing plants displayed a weaker growth with lower biomass and nitrate/nitrogen content (Figure 1), suggesting a negative regulation of ZmbHLH118 in response to nitrate. Notably, the finding that nitrate and nitrogen content decrease is more pronounced in roots (Figure 1E–H) indicates that ZmbHLH118 mainly functions in the root at the seedling stage. This is consistent with the higher expression level of *ZmbHLH118* in maize seedling root (Figure S4A). However, we did not detect nitrate and nitrogen content difference between *zmbhlh118‐ems* mutant and wild‐type plants (Figure S2). In maize, bHLH family consists of over 200 members [80], and our phylogenetic analysis showed that *ZmbHLH162*, *ZmbHLH164*, and *ZmbHLH172* are relatively close to *ZmbHLH118* (Figure S3B). Further experiments demonstrated that ZmbHLH162, ZmbHLH164, and ZmbHLH172 also repress the expression of LUC under the control of *ZmCLCa* promoter, like ZmbHLH118 (Figure S9), indicating a possible redundant function among these four bHLHs in regulating *ZmCLCa* gene expression. The existence of functional redundancy in bHLH family members in maize has been reported in several previous studies [64, 67, 69, 70, 92, 93, 94]. This functional redundancy can be the underlying reason for the lack of difference in the nitrate content in *zmbhlh118‐ems* single mutant (Figure S2). To further investigate the functions of these ZmbHLHs in regulating *ZmCLCa* and nitrate homeostasis in maize, higher‐order *zmbhlh* mutant plants are required in the future. Furthermore, post‐transcriptional and phosphorylation‐dependent signaling cascades also play an essential role in regulating nitrate/nitrogen transcriptional responses [90, 95]. Whether and how upstream protein kinases or ubiquitination enzymes regulate ZmbHLH118 in response to nitrate will also be a field of interest for future investigations.

In the past few decades, many vacuolar nitrate transporters have been identified and characterized, and CLCa is the most well‐studied one [26, 27, 28, 29, 30, 31, 33, 34, 42, 84]. However, the functional relevance and characterization of CLCa in crop plants are still unclear. In this study, we identified and characterized *CLCa* in maize plants. Subcellular localization experiments showed that ZmCLCa is localized in the tonoplast (Figures 5A and 7C; Figure S15A). Electrophysiological analyses showed that ZmCLCa mediates NO_3_^−^ transport across the vacuolar membrane and can catalyze NO_3_^−^ influx into the vacuole in a way comparable to AtCLCa (Figure 7D–F; Figure S15). This similar functional role between ZmCLCa and AtCLCa in regulating vacuolar NO_3_^−^ transport, nitrate/nitrogen content, and plant growth (Figures 3F and 6; Figures S12 and S13) most likely depends on the presence of a Proline residue in the selectivity filter conferring a preference for nitrate [32, 33, 38, 41]. Notably, our data showed that ZmCLCa is the only CLC family member in maize harboring this Proline residue, while in *Arabidopsis* it is present in AtCLCa and AtCLCb (Figure 3F). Notably, besides maize and *Arabidopsis*, this Proline residue in the selectivity filter of CLCa proteins is present in wheat, rice, and soybean (Figure S6), indicating that this Proline is evolutionarily conserved and is essential for CLCa selectivity for NO_3_^−^ between dicots and monocots.

Moreover, chlorate resistance assay on *zmclca* knock‐out plants and ZmbHLH118 overexpressing plants showed a dramatic decrease of chlorate resistance compared to the wild‐type plants (Figure S14), indicating that functional interruption (*zmclca‐1*, *zmclca‐2*) and expression defect (ZmbHLH118‐OE1, ZmbHLH118‐OE2) of ZmCLCa in maize plants will lead to an impairment of vacuolar loading for chlorate detoxification. The overaccumulation of chlorate in the cytoplasm results in enhanced chlorate sensitivity in maize plants (Figure S14), which further suggests a role of ZmCLCa in nitrate transport for vacuolar loading.

In *Arabidopsis*, AtCLCb is also localized in the tonoplast and facilitates nitrate transport from the vacuole to the cytoplasm under the condition of nitrate deficiency [42, 84]. Our phylogenetic analysis showed that *ZmCLCa* is the closest orthologous gene of both *AtCLCa* and *AtCLCb* in maize (Figure 3D). Although we have shown that ZmCLCa functions like AtCLCa in mediating NO_3_^−^ into the vacuole, we cannot exclude the possibility that ZmCLCa facilitates NO_3_^−^ efflux from the vacuole under certain conditions, like AtCLCb does in *Arabidopsis*. It is possible that ZmCLCa mediates NO_3_^−^ fluxes in both directions across the vacuolar membrane, depending on the conditions, mediating NO_3_^−^ influx into the vacuole to prevent NO_3_^−^ overaccumulation in the cytoplasm under surplus N conditions and releasing NO_3_^−^ from the vacuole to supply metabolism under N‐limited conditions. However, the data we obtained in the present study are rather in favor of a role of ZmCLCa in vacuolar NO_3_^−^ loading, which prevents NO_3_^−^ overaccumulation in the cytoplasm under surplus N conditions. The biophysical properties of ZmCLCa and its regulation by intracellular factors require further investigation.

Notably, the qRT‐PCR assay indicated that nitrate application transcriptionally triggered *ZmCLCa* expression (Figure 5E–G), which is consistent with the findings of *AtCLCa* in *Arabidopsis* [32]. However, in the past two decades, the upstream transcriptional regulator and the underlying mechanisms remained unknown. Here, we identified ZmbHLH118, a transcription factor that directly binds to the *ZmCLCa* promoter to suppress *ZmCLCa* expression (Figure 4). The inhibition of *ZmCLCa* by ZmbHLH118 overexpression and its upregulation by the functional disruption of ZmbHLH118 indicate that ZmbHLH118 negatively regulates *ZmCLCa* expression (Figure 4A,B). Among the ten E‐box motifs in the promoter of *ZmCLCa*, we identified two that were targets of ZmbHLH118 (Figure 4; Figure S8), negatively regulating the expression levels of *ZmCLCa* (Figure 4) and consequently modulating intracellular NO_3_^−^ homeostasis, nitrate/nitrogen content (Figure 6; Figures S12 and S13) and nitrate uptake (Figure 7A). It is possible that, in addition to ZmbHLH118 and its three closest homologs (ZmbHLH162, ZmbHLH164, and ZmbHLH172), other bHLH transcription factors can bind to the E‐box motifs and participate in the modulation of *ZmCLCa* expression in response to external nitrate. This could explain why *zmclca* mutant plants displayed a higher nitrate content reduction compared to the *zmbhlh118* mutant (Figure 6; Figures S2 and S12). Previous studies have shown that bHLH transcription factors regulate nitrogen metabolism, and *MhbHLH130* negatively regulated the expression of the chalcone synthase gene under low nitrate stress [91]. Our RNA‐seq analyses indicated that ZmbHLH118 also regulates genes involved in nitrogen and amino acid metabolism (Figure 3A; Figure S7) and provides an insight into how vacuolar nitrate storage regulation is integrated into nitrogen metabolism. Notably, our RNA‐seq analyses showed that ZmbHLH118 transcriptionally regulates multiple genes encoding ion channels and transporters in maize plants, including NPFs, ALMTs, EAD, and CLCs (Figure 3C). This indicates a potential broader role of ZmbHLH118 and its homologs in regulating cellular anion homeostasis, which needs further study.

Furthermore, disruption of ZmCLCa or overexpression of ZmbHLH118 leads to diminished chlorate resistance (Figure S14). In parallel, we also detected a weaker root uptake of nitrate in the *zmclca* mutant and ZmbHLH118 overexpressing plants (Figure 7A,B). These findings can be explained by the fact that the impairment of nitrate transport from cytoplasm to the vacuole leads to an overaccumulation of cytoplasmic nitrate, which triggers the systemic nitrate signaling to transcriptionally downregulate high‐affinity nitrate transporters at the plasma membrane and suppress the nitrate uptake by roots [19, 83]. Therefore, to modulate the cytoplasmic nitrate homeostasis, a crosstalk between the plasma membrane‐localized nitrate transporters/channels and the tonoplast‐localized nitrate transporters/channels may exist. The CBL‐CIPK network has been reported to regulate ion channels/transporters in the plasma and vacuolar membrane, modulating plant nutrient sensing and homeostasis [96]. At present, two main nitrate signaling pathways, NRT1.1‐CPK‐NLP and NRT1.1‐SPX‐NLP, are known to be involved in nitrate sensing and transduction [50, 95].

We found that nitrate‐induced transcriptional regulation of *ZmCLCa* and *ZmbHLH118* was impaired in *zmnrt1.1b* mutant plants (Figure S18A,B), suggesting that the plasma membrane‐localized NRT1.1‐mediated nitrate signaling modulates the ZmbHLH118‐ZmCLCa module to regulate vacuolar NO_3_^−^ transport process. However, the expression of *ZmCLCa* did not exhibit a difference between *zmnlp3* mutant and the wild‐type plants (Figure S19), indicating that the action of ZmbHLH118 on ZmCLCa is independent of NLP‐mediated nitrate signaling in maize.

The plasma membrane‐localized NRT1.1 and NRT2 transporters are responsible for nitrate uptake [30, 54, 56, 97, 98]. Our data showed that ZmbHLH118 influences the transcription levels of the plasma membrane‐localized nitrate transporter genes, including *ZmNRT1.1B* and *ZmNRT2.1*, in response to nitrate (Figure 3C; Figures S7A and S18C). This suggests a possible complex feedback regulation of ZmbHLH118 on plasma membrane‐mediated nitrate transport signaling, which will be of interest and require further investigation.

Overall, our study established a conceptual working model of a ZmbHLH118‐ZmCLCa module in regulating intracellular nitrate homeostasis and uptake in maize (Figure 8E). Functioning as a core tonoplast‐localized nitrate transporter in *Arabidopsis* and crop plants, the upstream regulatory and the underlying mechanisms of CLCa‐mediated NO_3_^−^ transport have been sought for over 20 years. Thus, in this study, identification of the ZmbHLH118‐ZmCLCa module assigns a novel understanding of the NO_3_^−^ transport process across the vacuolar membrane. Meanwhile, our study also provides two promising candidates, *ZmbHLH118* and *ZmCLCa*, for gene engineering to promote nitrogen use efficiency in maize. Although no significant differences were detected in grain nitrate contents, ZmbHLH118 and ZmCLCa transgenic plants displayed a significant change in the grain yield (Figure 8A–D; Figure S17). Thus, how to maximize the yield with higher NUE by genetic manipulation of ZmbHLH118 and ZmCLCa in agriculture is worthy of expectation.

## 4 Methods

### 4.1 Plant Materials and Growth Conditions

Maize (*Zea mays* L.) inbred line B73 was used for gene cloning and expression analyses. The transgenic lines, including overexpressing maize plants of ZmbHLH118 and all CRISPR/Cas9 mutants, including *zmclca*, *zmnrt1.1b*, and *zmnlp3.1* mutants, in the background of ND101, were generated by the Center for Crop Functional Genomics and Molecular Breeding (China Agricultural University, Beijing). Both ethyl methanesulfonate (EMS)‐induced point mutations of *zmbhlh118‐ems* and *zmclca‐ems* in the background of B73 were generated by the platform for maizeEMSDB of QiLu Normal University, Jinan [99]. We generated ZmCLCa overexpressing maize plants in the background of B104 at the Beijing BoMeiXingAo Technology Company, Beijing. A transgenic population harboring overexpression of maize transcription factor genes under the *Ubiquitin* promoter in the background of ND101 inbred line (wild type) was also generated by the Center for Crop Functional Genomics and Molecular Breeding (China Agricultural University). The primer sequences used to amplify and sequence are listed in Table S1.

To determine the phenotype of the maize transgenic plants grown under different nitrate conditions, the hydroponics experiments were carried out with a modified Hoagland solution for 2 weeks after 1‐week germination, then the shoot and root tissues were collected to measure the biomass, nitrate, and nitrogen content. The complete nutrient solution consisted of 0.1 mm KH_2_PO_4_, 0.6 mm MgSO_4_•7H_2_O, 1 mm K_2_SO_4_, 0.5 mm CaCl_2_•2H_2_O, 0.1 mm Fe(III)‐EDTA‐Na, 1 µm H_3_BO_3_, 0.5 µm MnSO_4_•H_2_O, 0.5 µm ZnSO_4_•7H_2_O, 0.2 µm CuSO_4_•5H_2_O and 0.07 µm Na_2_MoO_4_•2H_2_O. The N source was supplied in the form of KNO_3_ with indicated concentration (Low Nitrate, LN, 0.04 mm; Normal Nitrate, NN, 4 mm). The pH was adjusted to 5.8 with 1 m KOH. Maize seedlings were grown in an artificial climate chamber with 60% humidity at 28°C during the day and 22°C at night, under a 14‐h‐light and 10‐h‐dark photoperiod.

To investigate the dynamic transcriptional responses of *ZmbHLH118* and *ZmCLCa* to N fluctuation. The inbred line B73 was cultured under 2 mm NH_4_NO_3_ for 10 days and transferred to N‐free nutrient solution for 1, 2, or 4 days. Afterward, N was resupplied in the form of 4 mm KNO_3_ for 1, 3, 6, 12, 24, 48, or 72 h. Roots of maize plants were collected for qRT‐PCR.

For tissue‐specific expression pattern analysis of *ZmbHLH118* and *ZmCLCa* during the maize whole growth period, indicated tissues of B73 maize plants were collected for qRT‐PCR at the seedling stage, jointing stage, silking stage, and filling stage, respectively.

### 4.2 Measurement of Nitrate and Nitrogen Content

The nitrate content of the indicated samples was determined using the Plant Nitrate Nitrogen Content Assay Kit (ADS‐F‐N009, AIDISHENG, China) following the manufacturer's manual. In a nutshell, 0.1 g sample was boiled in 1 mL sterile water for 30 min, centrifuged to obtain 10 µL supernatant, and incubated with 40 µL 5% salicylic acid (dissolved in concentrated sulfuric acid) at room temperature for 10 min, adding 950 µL 8% sodium hydroxide, the reaction liquid became yellow, the 410 nm absorption peak was used to calculate the nitrate content.

The nitrogen content was determined using the samples that were dried at 65°C to constant weight, then ground into powder with a high‐flux ball mill. Each sample was weighted at about 0.1 g, and nitrogen content was determined by a carbon and nitrogen analyzer.

### 4.3 ^15^NO_3_^−^ Uptake Assay

One week after germination, maize seedlings were hydroponically grown under Hoagland nutrient solution supplied with 4 mm KNO_3_ for 10 days and then cultured 4 days with N‐free nutrient solution, followed by resupply of 4 mm KNO_3_ for 3 h. The plant roots were then rinsed in 1 mm CaSO_4_ for 1 min, then transferred to a Hoagland nutrient solution containing 4 mm K^15^NO_3_ (10 atom% ^15^N, Shanghai Research Institute of Chemical Industry) for 6 min ^15^N influx. Subsequently, roots were collected immediately after the final wash in CaSO_4_. Root samples were dried at 65°C to constant weight. Measurement of ^15^N content was performed by Delta V plus Isotope Mass Spectrometry (DELTA Plus XP, Thermo‐Finnigan, Germany). Uptake activity was calculated as the amount of ^15^N taken up per unit weight of roots per unit time.

### 4.4 RNA Extraction, cDNA Preparation, and qRT‐PCR

Total RNA was extracted from the indicated samples with RNA isolater Total RNA Extraction Reagent (R401, Vazyme, China). Approximately 1 µg total RNA was synthesized cDNA with HiScript III first Strand cDNA Synthesis Kit (R312, Vazyme, China). Then qRT‐PCR assays were performed with ChamQ Universal SYBR qPCR Master Mix (Q711, Vazyme, China) using Applied Biosystems QuantStudio 6 Flex. The transcript levels were obtained using 2^−ΔΔCt^ method based on *ZmTUB*. The primers used for qRT‐PCR are listed in Table S1.

### 4.5 RNA‐seq Assays

Wild‐type (ND101) and overexpressing maize plants of ZmbHLH118 (ZmbHLH118‐OE1) were grown under LN and NN conditions for 2 weeks, and root tissues were collected (three biological replicates). Total RNA was extracted, and mRNA libraries were sequenced on an Illumina Novaseq platform at Novogene (Beijing), and 6 GB clean data were generated. The reads were mapped to the maize B73 reference genome (B73 RefGen_v4, AGPv4) using HISAT2 (v.2.0.5) with default parameters. Differentially expressed genes (DEGs) analysis of wild type and ZmbHLH118‐OE1 under LN and NN conditions was performed using the DESeq2 R package. Corrected FDR < 0.005 was set to clarify genes that were significantly and differentially expressed. Using the NovoMagic (https://magic‐plus.novogene.com) for data analysis and chart generation, using maizeGDB (https://www.maizegdb.org/) for gene annotation, using MEGA11 for the generation of evolutionary trees, and using DNAMAN for amino acid sequence comparison.

### 4.6 Intracellular Localization Assay

The protoplasts of maize mesophyll cells transiently overexpressing ZmbHLH118‐GFP and ZmCLCa‐GFP were digested by Cellulase R10 (Yakult Honsha, Japan) and Macerozyme R10 (Yakult Honsha, Japan). The protoplasts of *Nicotiana benthamiana* and *Arabidopsis* mesophyll cells transiently overexpressing ZmCLCa‐GFP were digested by cellulase R‐10 (Yakult Honsha, Japan) and pectolyase Y‐23 (Yakult Honsha, Japan). The fluorescence signals of maize and tobacco protoplasts were examined using Zeiss Laser Scanning Confocal Microscope (LSM880, Germany), and those of *Arabidopisis* were examined using Leica STELLARIS 8 (Leica, Germany). The nuclear dye, DAPI solution, was used to counterstain the nucleus (28718‐90‐3, Solarbio, China). The maize and tobacco vacuoles were released using an osmotic shock buffer containing: 0.1 mm CaCl_2_, 3 mm MgCl_2_, 100 mm HCl, moderate BisTrisPropane to pH 7.5, 500 mOsm, and the *Arabidopsis* vacuoles were released using the buffer containing: 100 mm DL‐malic acid, pH 6 with BisTrisPropane, osmolarity fixed at 550 mOsm with sorbitol.

### 4.7 Histochemical Localization of β‐Glucuronidase (GUS) Expression

The 3‐kb promoter of *ZmCLCa* was cloned into the pCXUN::GUS vector [100] to generate the *proZmCLCa*‐GUS transgenic maize plants. The root tissues of a 7‐day‐old transgenic maize plant were stained at 37°C using a GUS staining kit (SL7160, Coolaber, China). The blue colored tissues were embedded in Tissue‐Tek O.C.T. Compound (SAKURA, America), and then were frozen and sectioned using a microtome cryostat (Leica CM1850, Germany), and were imaged under a Nikon ECLIPSE Ti2 microscope (Nikon, Japan).

### 4.8 Chlorate Resistance Assay

Determination of chlorate (ClO_3_^−^) resistance was investigated by employing transgenic maize seedlings after 1‐week germination. Maize seedlings were cultured in 0 mm or 4 mm potassium chlorate (KClO_3_) solution, and the solution was changed every 2 days. Seedlings were usually grown in an artificial climate chamber with 60% humidity at 28°C during the day and 22°C at night, under a 14‐h‐light and 10‐h‐dark photoperiod. The shoot fresh weight of maize seedlings was measured when toxic phenotypes appeared between wild‐type and transgenic maize seedlings. Chlorate resistance was calculated as the following equation according to Teng et al. [87] and Gao et al. [17]: Chlorate resistance (%) = (shoot fresh weight in 4 mm ClO_3_^−^ / shoot fresh weight in free ClO_3_^−^) × 100.

### 4.9 Transcriptional Activity Assays

For transcriptional activation or inhibition activity assay of ZmbHLH118, the yeast strain Y2HGold (*Saccharomyces cerevisiae*) was transformed with the corresponding bait vectors. The full‐length coding sequence of *ZmbHLH118* was inserted into vectors pGBKT7 and pGBKT7‐VP16, respectively. Vector pGBKT7 containing the GAL4 DNA‐binding domain without the activation domain was used as a negative control, and vector pGBKT7‐VP16 containing the GAL4 DNA‐binding domain and activation domain was used as a positive control. Transformants were screened on SD/‐Trp and SD/‐Trp‐His‐Ade medium. The initial concentration of yeast was modulated to an OD_600_ value of 0.2 and diluted to 1/10, 1/100, and 1/1000 according to the gradient, before incubating the plates at 28°C for 3 days.

### 4.10 Electrophysiological Assay

The *clca‐3* knockout line employed in this experiment matches Gabi Kat GK‐624E03‐022319 using the Colombia (Col‐0) ecotype as the genetic background [24]. BisTrisPropane (BTP) was employed as an impermeable cation to isolate anion currents [33]. Currents were recorded using an EPC‐10 amplifier (HEKA Electronics, Lambrecht/Pflaz, Germany). Data were acquired and analysed using PATCH MASTER and FIT MASTER software (HEKA). The vacuolar side buffer contained: (for 200 mm NO_3_^−^) 200 mm HNO_3_, 100 mm BTP, 1 mm CaCl_2_, 5 mm MES, pH 5.5 adjusted with MES, the osmolarity was fixed at 550 mOsm with sorbitol. The cytosolic side buffer contained: (for 19.2 mm Cl^−^) 15 mm HCl, 7.5 mm BTP, 0.1 mm CaCl_2_, 2 mm MgCl_2_, 15 mm MES, pH 7 adjusted with BTP, the osmolarity was fixed at 500 mOsm with sorbitol. Currents were recorded using the voltage‐pulse protocol: starting and ending at the holding potential of −19 mV for 1 s, voltage pulses were applied starting from −79 to +41 mV following +20 mV increments, and a post‐pulse was applied after each pulse at −69 mV. Currents were recorded using a sampling interval of 1 ms. The liquid junction potential measured was −19 mV and was corrected accordingly [101].

Currents detected using *Nicotiana benthamiana* were performed in the whole‐vacuole configuration and recorded with an Axon 200 B patch‐clamp amplifier (MD Institutions, San Jose, CA, USA) using an Axon Digidate 1550 B (MD Institutions). The recordings were acquired and analyzed with Clampfit 11 software (MD Institutions). Nitrate cytosolic solutions contained: (for 4.2 mm NO_3_^−^) 0.1 mm Ca(NO_3_)_2_, 2 mm Mg(NO_3_)_2_, 15 mm MES, pH 7 with BTP, the osmolarity was fixed at 500 mOsm with sorbitol. Nitrate vacuolar solutions contained: (for 100 mm NO_3_^−^) 100 mm HNO_3_, 5 mm HCl, pH 5.5 with BTP, the osmolarity was fixed at 550 mOsm with sorbitol. Currents were recorded using the voltage‐pulse protocol: starting and ending at the holding potential of 0 mV, voltage pulses were applied starting from −120 to +60 mV, following +20 mV increments, and a post‐pulse was applied after each pulse at −40 mV. Currents were recorded using a sampling interval of 0.1 ms. The potentials were corrected by the liquid junction potential [101].

The vacuole release and the cytosolic side buffers were exchanged using a gravity‐driven perfusion system, and the analysis was done on recordings at 10 min after the whole vacuole configuration was obtained to ensure the dialysis of the vacuolar lumen.

### 4.11 Dual Luciferase Reporter Assay

A dual luciferase reporter assay was performed using *Nicotiana benthamiana*. The full‐length promoter (1.5‐kb) sequence of *ZmCLCa* was cloned into the pCAMBIA1302‐LUC vector to obtain fusion vector 35S::REN::*proZmCLCa*::LUC, and the coding sequence of *ZmbHLH118* was cloned into the 35S::GFP vector with corresponding primers (Table S1). The fusion vectors were transformed into *Agrobacterium tumefaciens* strain GV3101 and infiltrated into tobacco leaves in specified combinations. Images of the LUC signal were obtained using a Tanon 5200 Multi Chemiluminescent Imaging System (Tanon, China), and the luciferase intensity was calculated by ImageJ of at least five biological replicates.

### 4.12 Yeast One‐Hybrid (YIH) Assay

To perform the yeast one‐hybrid assay, the full‐length promoter (1.5‐kb) and several promoter fragments containing the E‐box of *ZmCLCa* were cloned into the pAbAi vector; linearized recombinant vectors were integrated into the yeast strain YIHGold (*Saccharomyces cerevisiae*), and the coding sequence of *ZmbHLH118* was cloned into the pGADT7 vector. The primers are listed in Table S1. The transformed yeast cells were screened on SD/‐Leu with the indicated Aureobasidin A (AbA) concentration. The initial concentration of yeast was modulated to an OD_600_ value of 0.2 and diluted to 1/10, 1/100, and 1/1000 according to the gradient, before incubating the plates at 28°C for 3 days.

### 4.13 Chromatin Immunoprecipitation‐Quantitative PCR (ChIP‐qPCR) Assay

A ChIP‐qPCR assay was performed using maize protoplasts of inbred line B73. The coding sequence of *ZmbHLH118* was cloned into the 35S::GFP vector. The empty GFP vector and the fusion vector harboring ZmbHLH118 were transiently transformed into maize protoplasts. The chromatin was sheared by ultrasonic crushing to obtain DNA fragments using Bioruptor Plus (Diagenode, Belgium). The fragmented DNA of the *ZmCLCa* promoter pulled down by ZmbHLH118‐GFP was purified with QIAquick PCR Purification Kit (QIAGEN, Germany) and then used for qRT‐PCR assay. Fold enrichment was calculated based on the ratio of immunoprecipitation to input. The primers are listed in Table S1.

### 4.14 Electrophoretic Mobility Shift Assay (EMSA)

The coding sequence of *ZmbHLH118* was amplified and cloned into the pMAL‐c5X vector to generate ZmbHLH118‐MBP fusion vector. The proteins were purified and used to electrophoretic mobility shift assay. The two 40‐bp promoter fragments containing E‐box binding motif of the *ZmCLCa* were labeled with 5′‐end biotin, which was synthesized by Beijing RuiBiotech as DNA probes; unlabeled sequences were used as the competitive probes (Cold‐Probe). EMSA assay was performed using the LightShift Chemiluminescent EMSA Kit (GS009, Beyotime, China) according to the manufacturer's protocols. The primers are listed in Table S1.

### 4.15 Statistical Analyses

All statistical analyses were performed using GraphPad Prism 8.0.2 software. Each treatment was performed with at least 3 biological or technical replicates, and the statistical parameters, such as the means ± SD (Standard Deviation) and means ± SEM (Standard Error of the Mean), are shown in the corresponding figure legend. Statistical significance was assessed with a two‐tailed Student's *t*‐test for comparisons between two groups, and with one‐way analysis of variance (ANOVA) followed by Tukey's multiple comparison test for comparisons among multiple groups. Significance was denoted by either letters or asterisks. Different letters indicate statistical significance at *p* < 0.05 (the control group was labelled a). Asterisks indicate statistical significance, * *p* < 0.05, ** *p* < 0.01, *** *p* < 0.001, and ns represents no significant (*p* > 0.05). The specific statistical analyses were conducted as described in the corresponding figure legend.

### 4.16 Accession Numbers

Sequences data used in this article were deposited with MaizeGDB (https://www.maizegdb.org/) and TAIR (https://www.arabidopsis.org/) under the following accession numbers: ZmbHLH118, GRMZM2G061906; *ZmbHLH162*, GRMZM2G093744; *ZmbHLH164*, GRMZM2G058451; *ZmbHLH172*, GRMZM2G017586; *ZmCLCa*, Zm00001d046919; *ZmCLCc*, Zm00001d042651; *ZmCLCd*, Zm00001d033597; *ZmCLCe*, Zm00001d043721; *ZmCLCf*, Zm00001d017868; *ZmCLCg1*, Zm00001d002091; *ZmCLCg2*, Zm00001d015702; *AtCLCa*, AT5G40890; *AtCLCb*, AT3G27170; *AtCLCc*, AT5G49890; *AtCLCd*, AT5G26240; *AtCLCe*, AT4G35440; *AtCLCg*, AT5G3322280; *AtCLCf*, AT1G55620. The sequences of other bHLH family was acquired in PlantTFDB (https://planttfdb.gao‐lab.org/index.php).

## Author Contributions

C.Z. performed most of the experiments and analyzed the data; E.D.C. performed patch‐clamp experiments in *Arabidopsis*; X.L. participated in the field experiment; Z.G., H.Z., and H.C. participated in the maize physiological measurement; Y.S., L.M., K.Z., J.Z., Z.J., and L.Y. participated in the data analysis; C.Z., A.D.A., and J.B.Z. wrote and revised the manuscript. All authors read and approved the manuscript. J.B.Z. conceived the whole project.

## Funding

This research was supported by the National Key Research and Development Program of China (2021YFF1000501 and 2022YFD1900704 to J.B.Z.), National Natural Science Foundation of China (32070306 to J.B.Z.), the 2115 Talent Development Program of China Agricultural University (to J.B.Z.), the French National Research Agency (ANR‐10‐INBS‐04 to A.D.A.), and the Hainan Provincial Natural Science Foundation of China (323CXTD379 to J.Z.).

## Conflicts of Interest

The authors declare no conflicts of interest.

## Supporting information




**Supporting File**: advs74498‐sup‐0001‐SuppMat.doc.

## Data Availability

Research data are not shared.
