# Supplementary material for: The Maize ZmbHLH118 Transcription Factor Regulates Vacuolar Nitrate Loading by the NO3 − Transporter ZmCLCa
Source: Adv Sci (Weinh). 2026 Feb 21;13(25):e20219. doi: 10.1002/advs.202520219 (PMC13137819; doi:10.1002/advs.202520219)
Supplement: Supplementary file 1 — Supporting File: advs74498‐sup‐0001‐SuppMat.doc. [file ADVS-13-e20219-s001.doc]

Supporting Information

**The Maize ZmbHLH118 Transcription Factor Regulates Vacuolar Nitrate Loading by the NO_3_^-^ Transporter ZmCLCa**

*Chaonan Zhang, Elsa Demes-Causse, Xujian Li, Zhenhui Guo, Hanshu Zhao, Huairong Cao, Yajing Song, Lijun Mu, Kaikai Zhang, Jing Zhang, Zhongtao Jia, Lixing Yuan, Alexis De Angeli^*^, Jingbo Zhang^*^*

*E-mail: jingbozhang@cau.edu.cn; alexis.deangeli@cnrs.fr

**List of Supporting Information**

**Figure S1.** The transcript levels of *ZmbHLH118* in ZmbHLH118 overexpressing maize plants.

**Figure S2.** ZmbHLH118 negatively regulates maize growth.

**Figure S3.** Phylogenetic trees of the ZmbHLH family.

**Figure S4.** The *ZmbHLH118* expression across different tissues of maize plants.

**Figure S5.** Transcriptomics analysis of ZmbHLH118 overexpressing and wild-type maize plants under NN and LN conditions.

**Figure S6.** Alignment of the Proline 160 amino acid residue in wheat, rice, and soybean.

**Figure S7.** ZmbHLH118 negatively regulates the expression of *ZmNRT2.1*, *ZmNR1.1* and *ZmNIR1.1* in maize.

**Figure S8.** ZmbHLH118 cannot directly bind to the other promoter fragments of *ZmCLCa* except P3.

**Figure S9.** ZmbHLHs negatively regulate the expression of *ZmCLCa* in maize.

**Figure S10.** The *ZmCLCa* expression across different tissues of maize plants.

**Figure S11.** Generation of *zmclca* knockout mutants.

**Figure S12.** ZmCLCa positively regulates maize growth and nitrate content.

**Figure S13.** Overexpression of ZmCLCa promotes maize growth.

**Figure S14.** ZmCLCa promotes chlorate resistance in maize.

**Figure S15.** ZmCLCa mediates NO_3_^-^ transport into the vacuole.

**Figure S16.** Plant height of ZmbHLH118 and ZmCLCa transgenic maize plants in the field.

**Figure S17.** Function-loss of ZmbHLH118 promotes maize growth and yield in the field.

**Figure S18.** The mutual regulation of expression levels between *ZmNRT1.1B* and *ZmbHLH118*.

**Figure S19.** ZmNLP3.1 does not regulate *ZmCLCa* expression.

**Table S1.** List of primers used in this study.


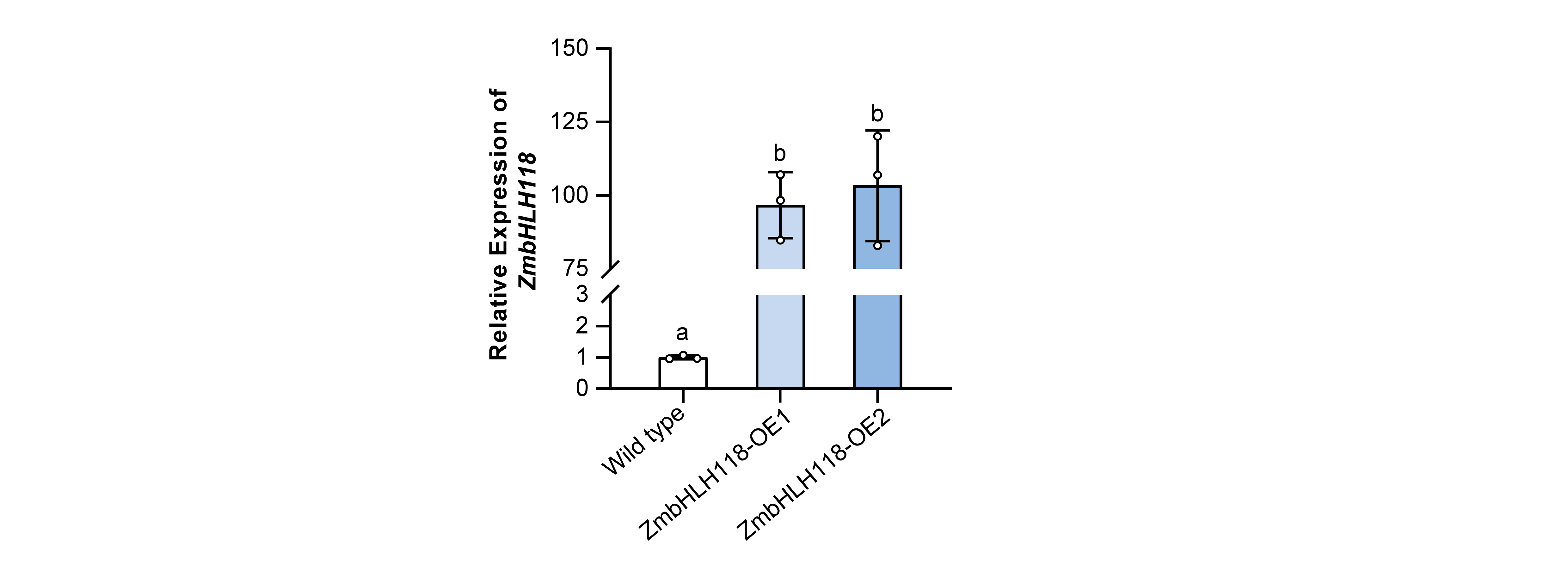


**Figure S1. The transcript levels of *ZmbHLH118* in ZmbHLH118 overexpressing maize plants.** The transcript levels of *ZmbHLH118* in wild-type and ZmbHLH118 overexpressing maize plants. RNA samples were prepared using two-leaf-old maize seedlings, and the qRT-PCR assay was performed, *ZmTUB* was used as the internal reference and the transcript levels of wild type was set to 1. Data are means ± SD (*n* = 3 technical replicates). Statistical significance was determined using one-way ANOVA followed by Tukey’s multiple comparison test. Different letters represent a significant difference at *p* < 0.05.


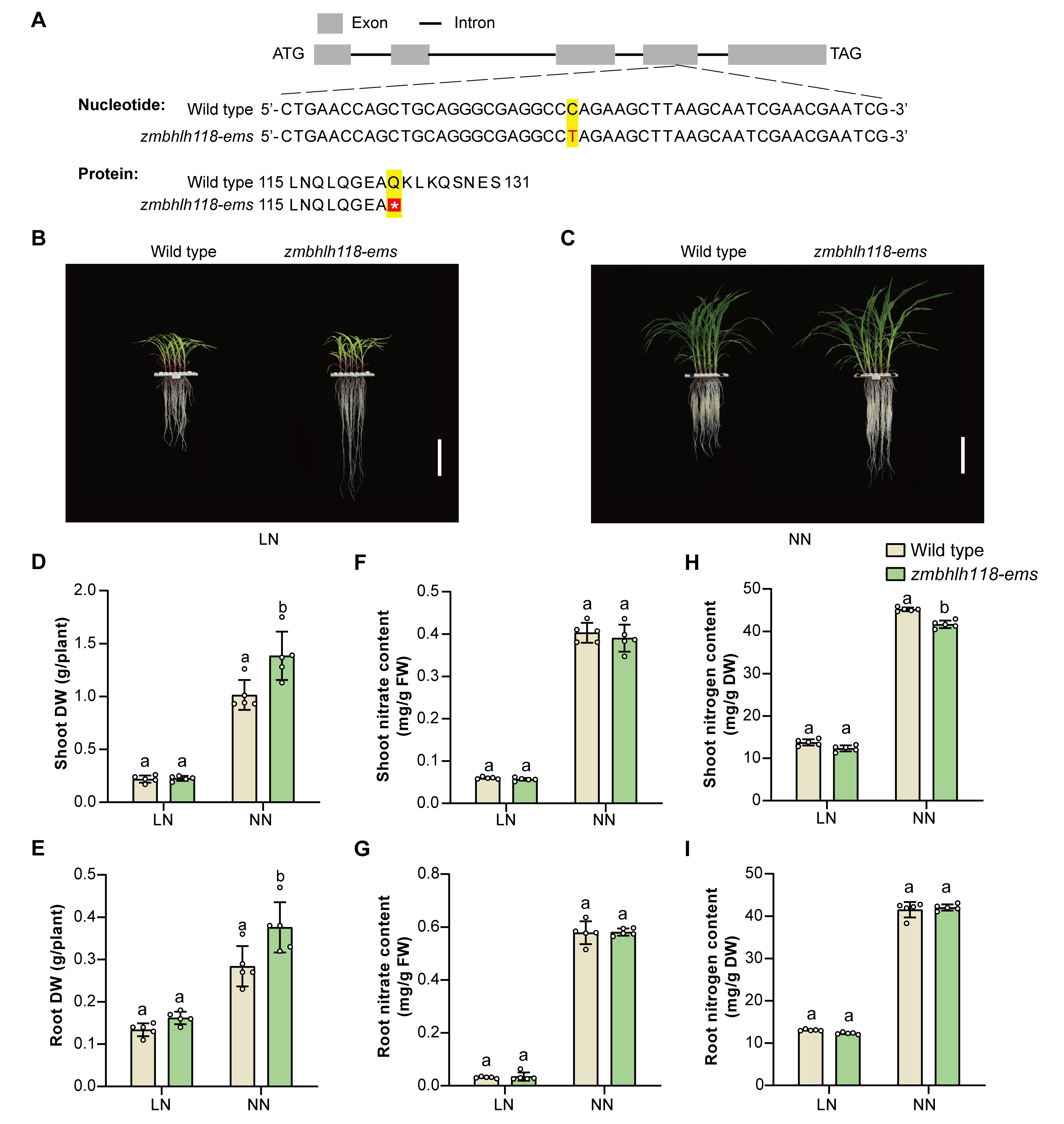


**Figure S2.** **ZmbHLH118 negatively regulates maize growth.** (**A**) Generation of *zmbhlh118-ems* mutant. Alignment of the nucleotide sequences and the protein sequences of wild type and *zmbhlh118-ems* are shown, the mutation sites are colored in red with yellow highlight,
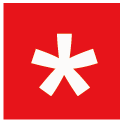
 indicates the stop codon. Three-week-old *zmbhlh118* EMS mutant (*zmbhlh118-ems*) and wild-type maize plants growth under (**B**) Low Nitrate (LN, 0.04 mm KNO_3_) and (**C**) Normal Nitrate (NN, 4 mm KNO_3_) conditions. (**D**, **E**) The biomass, (**F**, **G**) nitrate concentration and (**H**, **I**) nitrogen concentration in shoot and root tissue of *zmbhlh118-ems* and wild-type maize plants grown in indicated nitrate conditions. DW, dry weight; FW, fresh weight. (**B** and **C**) Scale bars, 20 cm. Data in (**D**-**I**) are means ± SD (*n* = 5 biological replicates). Statistical significance was determined using two-tailed Student’s *t*-test. Different letters represent a significant difference at *p* < 0.05.


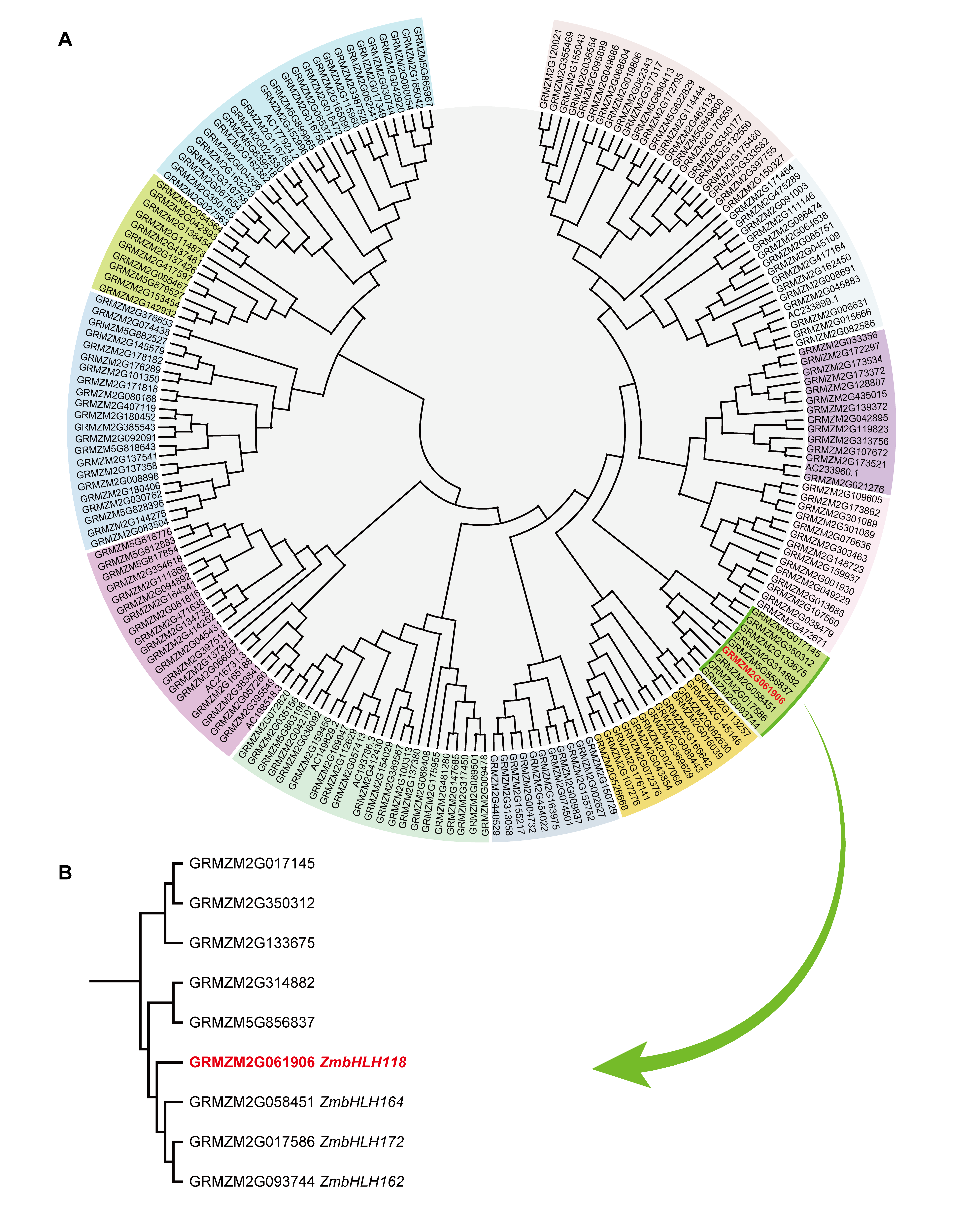


**Figure S3. Phylogenetic trees of the ZmbHLH family.** (**A**) The evolutionary tree of 208 genes of ZmbHLH family was generated using MEGA11. (**B**) A detailed diagram of the branch where *ZmbHLH118* was located.


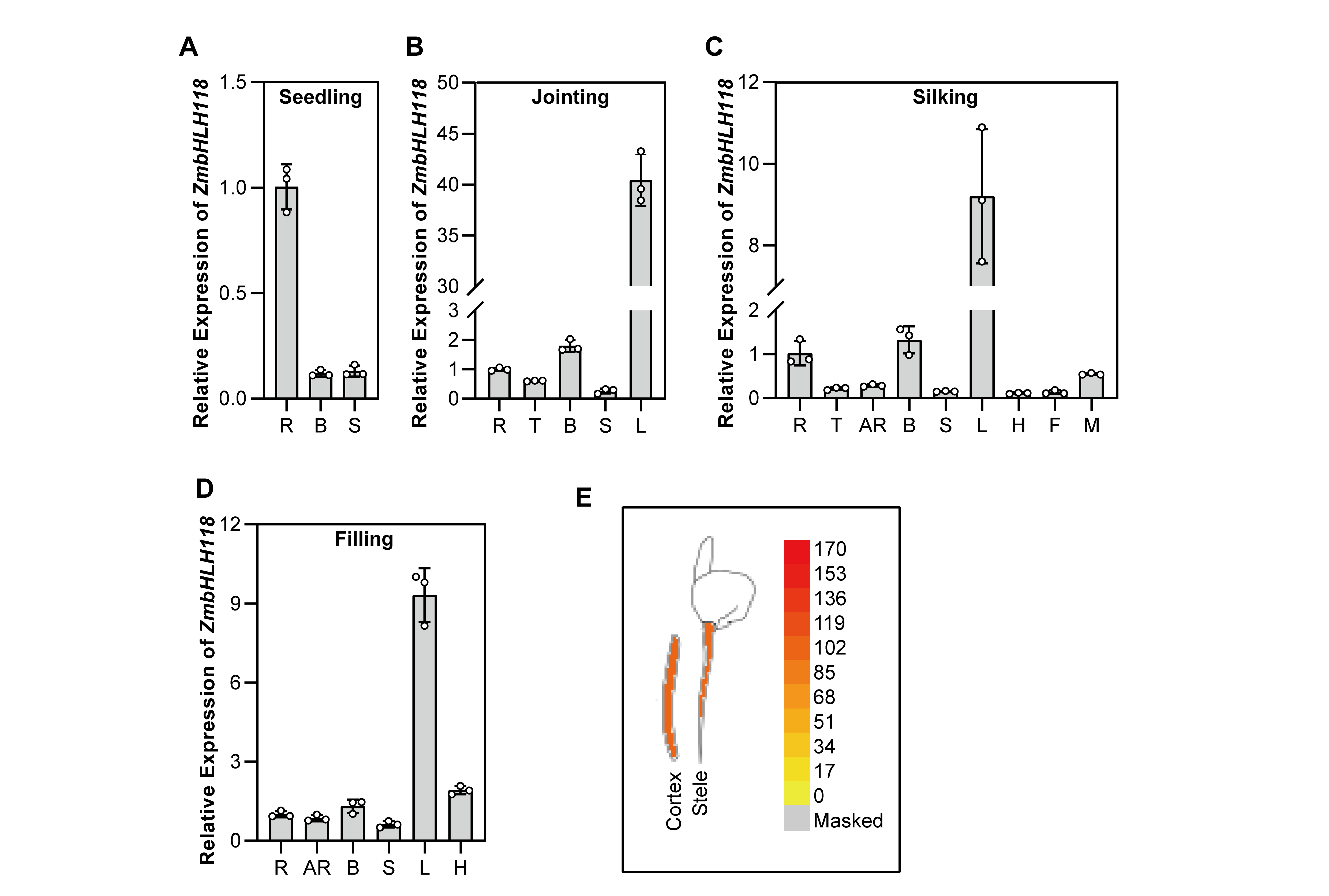


**Figure S4. The *ZmbHLH118* expression across different tissues of maize plants.** The transcript levels of *ZmbHLH118* across different tissues at (**A**) seedling stage, (**B**) jointing stage, (**C**) silking stage and (**D**) filling stage were detected by qRT-PCR. R, root; T, root tip; A, air root; B, basal; S, shoot (seedling stage) /stem; L, leaf; H, husk; F, female spike; M, male spike. (**E**) The heat map shows the transcript levels of *ZmbHLH118* in the root cortex and stele tissues. The figure was modified from [https://www.maizegdb.org.](https://www.maizegdb.org./) *ZmTUB* was used as the internal reference and the expression level of *ZmbHLH118* in the root was set to 1. Data in (**A**-**D**) are means ± SD (*n* = 3 technical replicates).


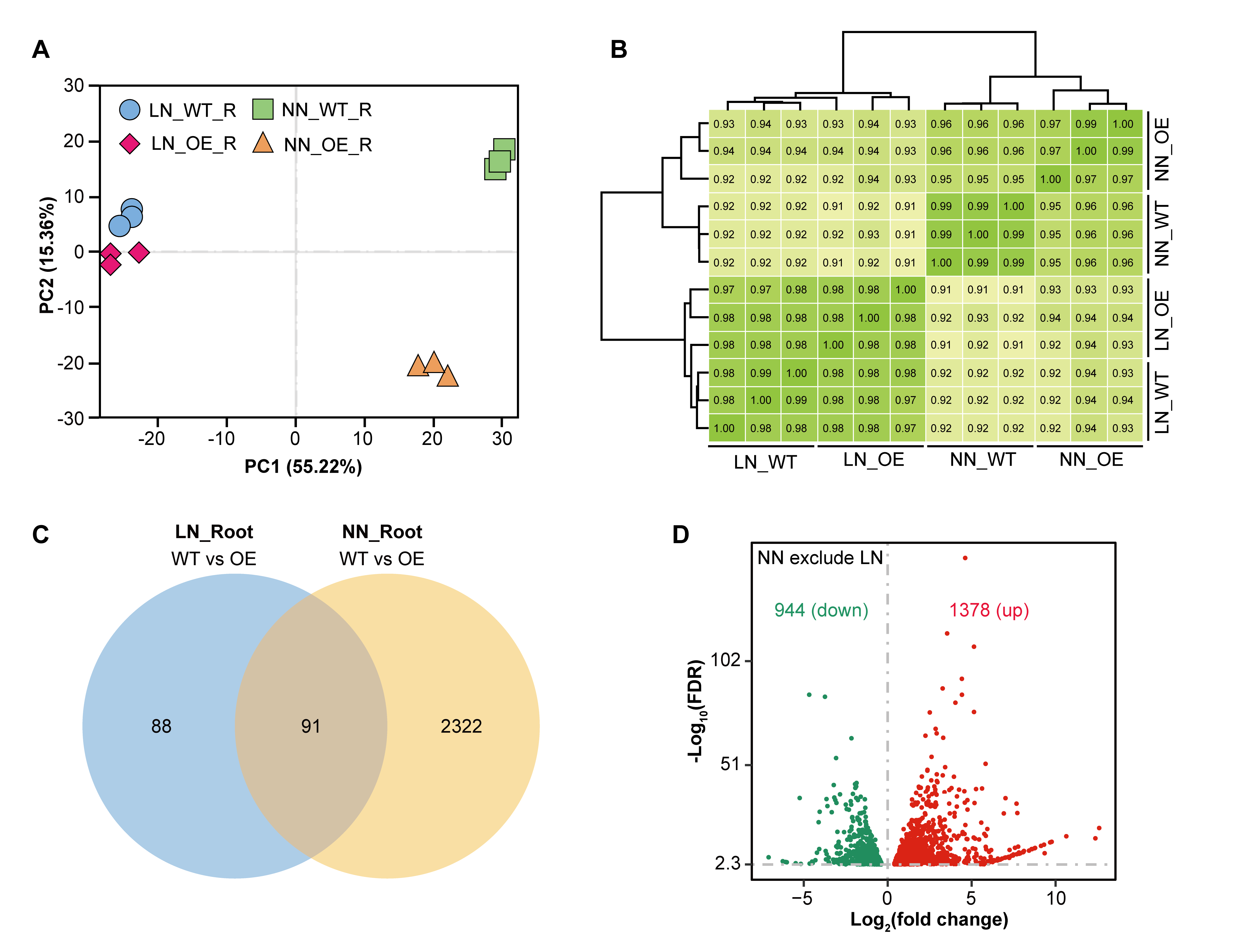


**Figure S5. Transcriptomics analysis of ZmbHLH118 overexpressing and wild**-**type maize plants under NN and LN conditions.** Transcriptomics on roots of three-week-old ZmbHLH118-OE and wild-type maize plants treated with Low Nitrate (LN, 0.04 mm KNO_3_) or Normal Nitrate (NN, 4 mm KNO_3_). (**A**) Principal component analysis and (**B**) Correlation heat map analysis showed a high degree of consistency among repetitions. (**C**) Venn diagram showed the overlap between wild-type and ZmbHLH118-OE plants under Low Nitrate (LN, 0.04 mm KNO_3_) and Normal Nitrate (NN, 4 mm KNO_3_). (**D**) Volcano plots showed the change of 2322 differentially expressed genes (DEGs) in the root tissue of wild-type and ZmbHLH118-OE plants under NN exclude LN. DEGs were identified using FDR < 0.005 as a criterion.


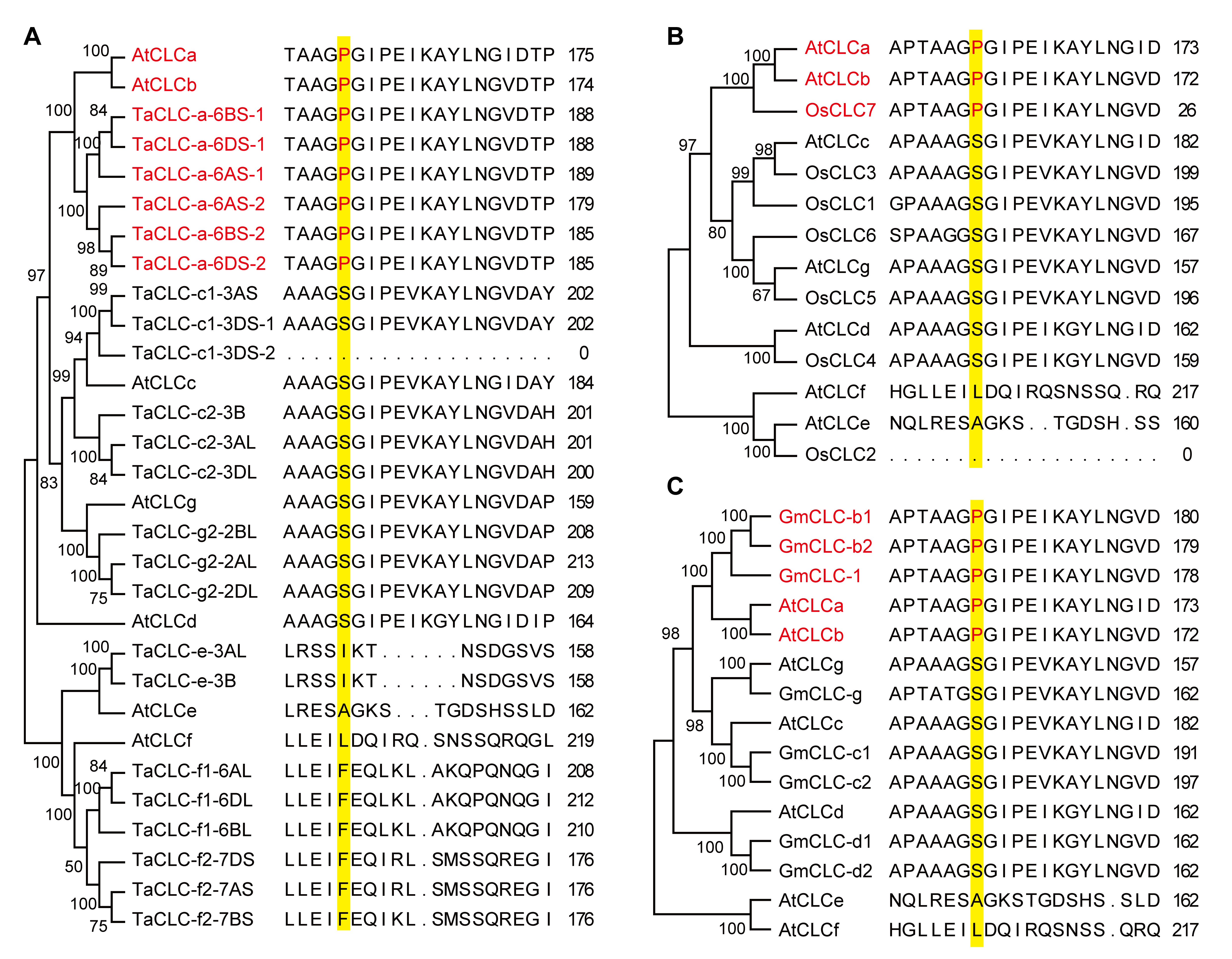


**Figure S6. Alignment of the Proline 160 amino acid residue in wheat, rice, and soybean.** Phylogenetic trees and alignment of the Proline 160 amino acid residue of wheat (**A**), rice (**B**) and soybean (**C**). The corresponding amino acid is highlighted in yellow, and Proline is colored in red.


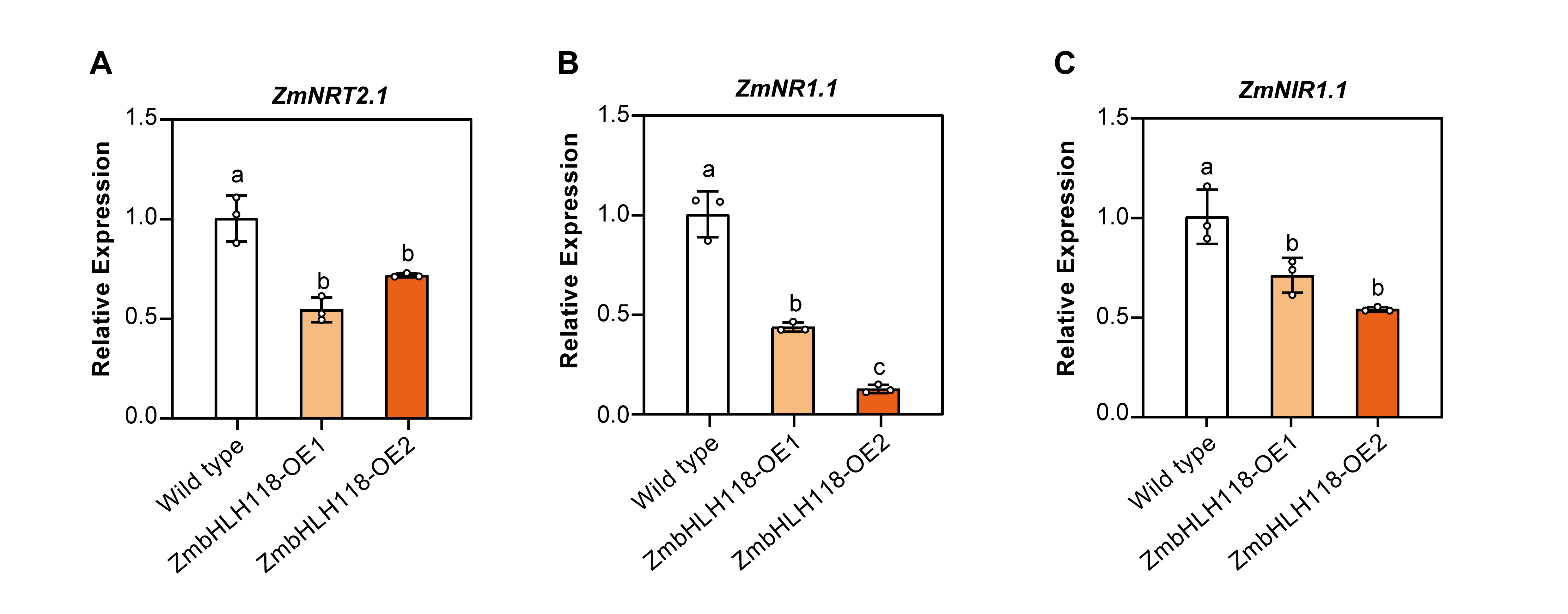


**Figure S7. ZmbHLH118 negatively regulates the expression of *ZmNRT2.1*, *ZmNR1.1* and *ZmNIR1.1* in maize.** The transcript levels of *ZmNRT2.1* (**A**), *ZmNR1.1* (**B**) and *ZmNIR1.1* (**C**) in wild-type and ZmbHLH118 overexpressing maize plants under NN conditions. The nitrate uptake and assimilation related genes in root tissues of three-week-old maize plants were determined by qRT-PCR analysis. *ZmTUB* was used as the internal reference and the expression level in the wild type was set to 1. Data in (**A**-**C**) are means ± SD (*n* = 3 technical replicates). Statistical significance was determined using one-way ANOVA followed by Tukey’s multiple comparison test. Different letters represent a significant difference at *p* < 0.05.


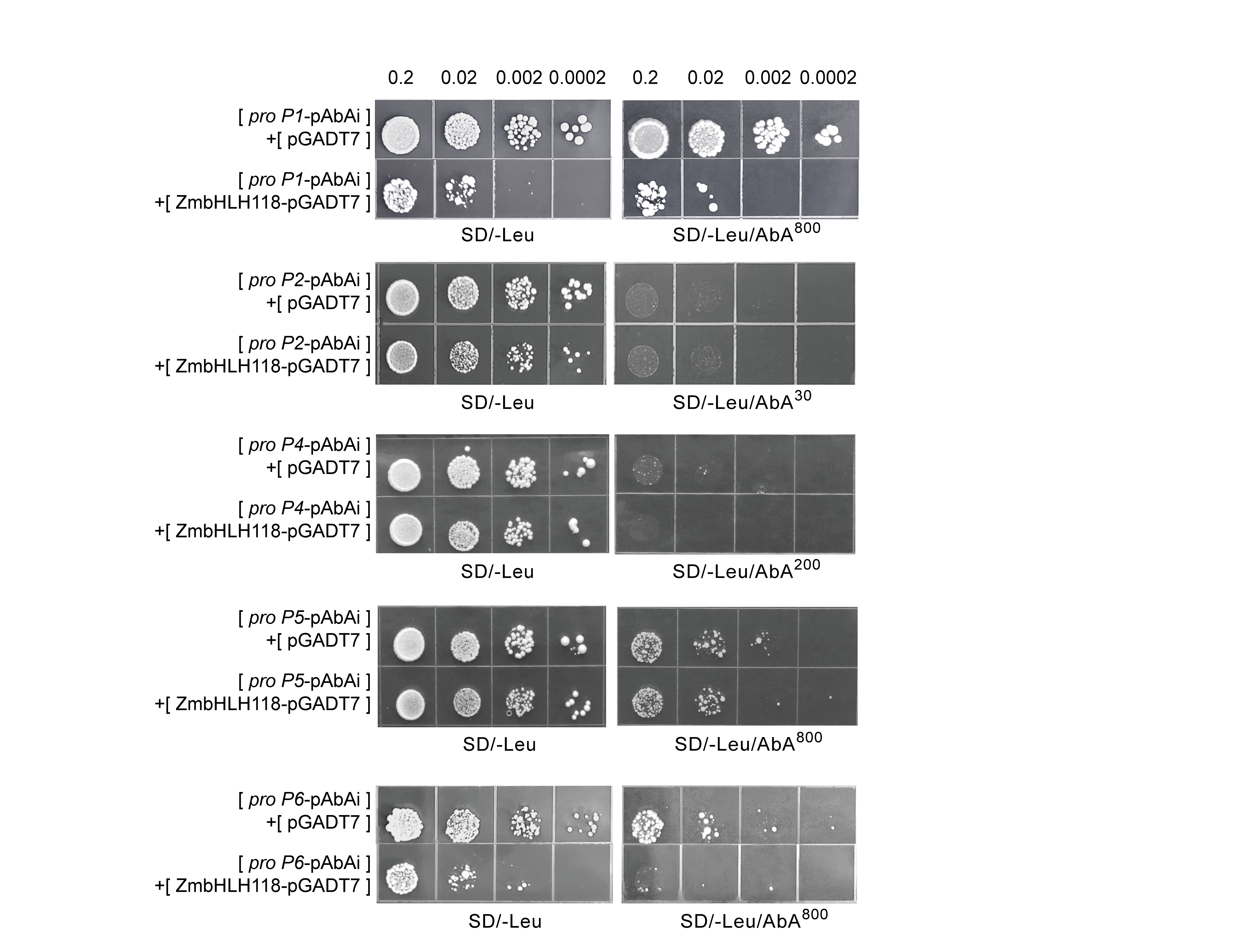


**Figure S8. ZmbHLH118 cannot directly bind to the other promoter fragments of *ZmCLCa* except P3.** ZmbHLH118 cannot directly bind the P1, P2, P4, P5, P6 promoter fragments of *ZmCLCa* by Y1H assay. The transformants were screened on SD/-Leu media containing indicated Aureobasidin A (AbA) conditions.

**
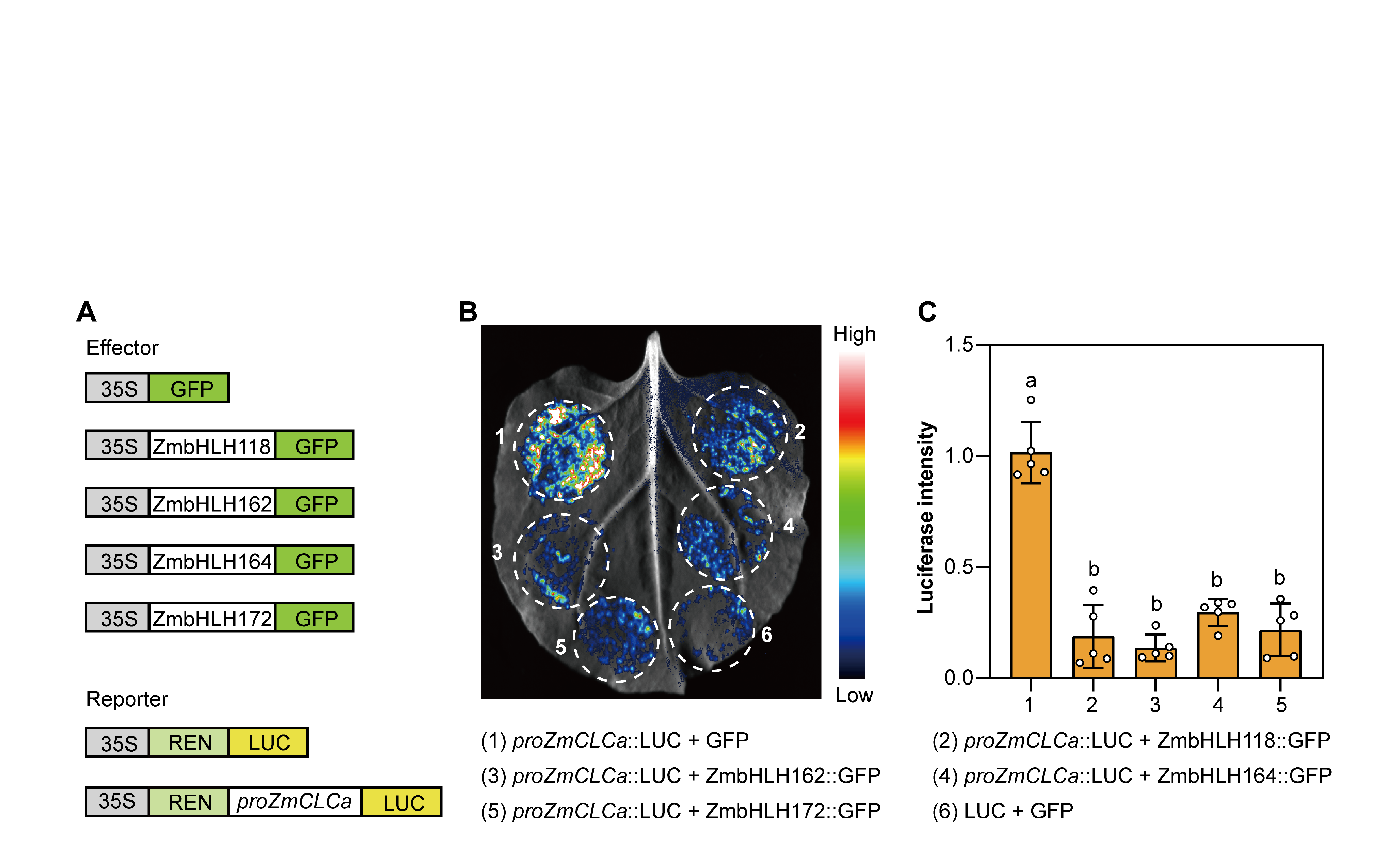
**

**Figure S9. ZmbHLHs negatively regulate the expression of *ZmCLCa* in maize.** (**A**) Schematic diagram of the reporter and effector constructs used in the dual luciferase assay. (**B**) The appearance of dual luciferase assay and (**C**) the relative luciferase intensity of the *ZmCLCa* promoter co-expressing with ZmbHLH118 (2), ZmbHLH162 (3), ZmbHLH164 (4), and ZmbHLH172 (5) in *Nicotiana benthamiana*, respectively. The luciferase intensity was calculated by ImageJ of five biological replicates. The luciferase intensity of *proZmCLCa*::LUC + GFP was set to 1. Data in (**C**) are means ± SD (*n* = 5 biological replicates). Statistical significance was determined using one-way ANOVA followed by Tukey’s multiple comparison test. Different letters represent a significant difference at *p* < 0.05.


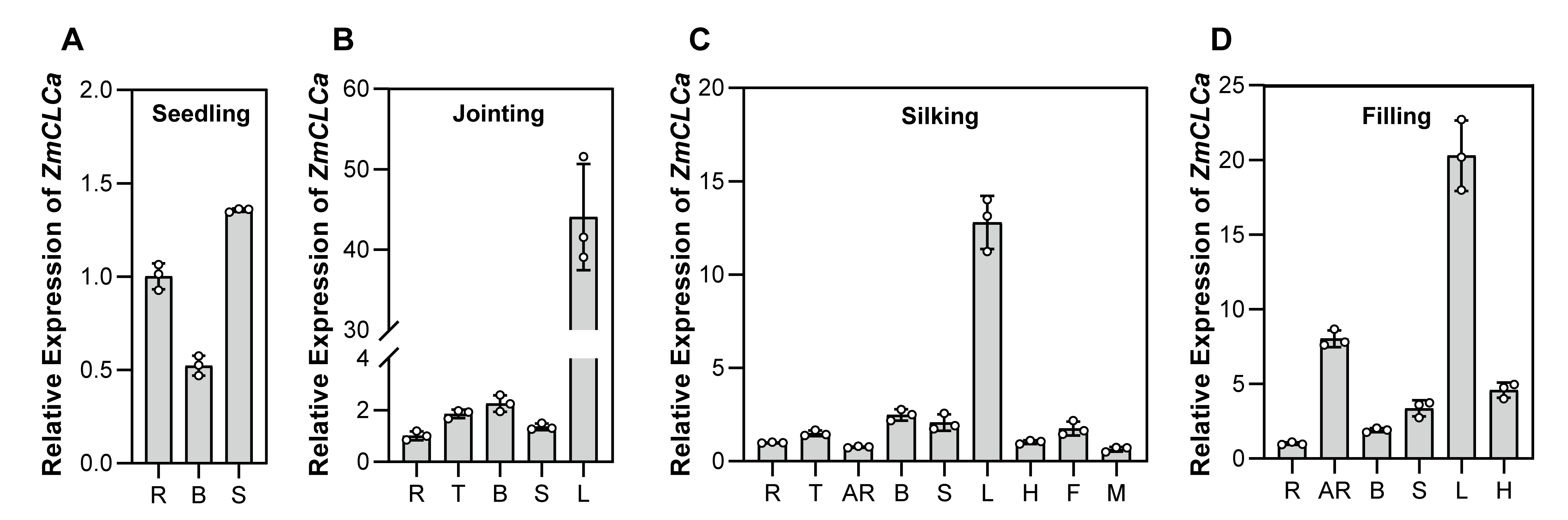


**Figure S10.** **The *ZmCLCa* expression across different tissues of maize plants.** The transcript levels of *ZmCLCa* across different tissues at (**A**) seedling stage, (**B**) jointing stage, (**C**) silking stage and (**D**) filling stage were detected by qRT-PCR. R, root; T, root tip; A, air root; B, basal; S, shoot (seedling stage) /stem; L, leaf; H, husk; F, female spike; M, male spike. *ZmTUB* was used as the internal reference and the expression level of *ZmCLCa* in the root was set to 1. Data in (**A**-**D**) are means ± SD (*n* = 3 technical replicates).


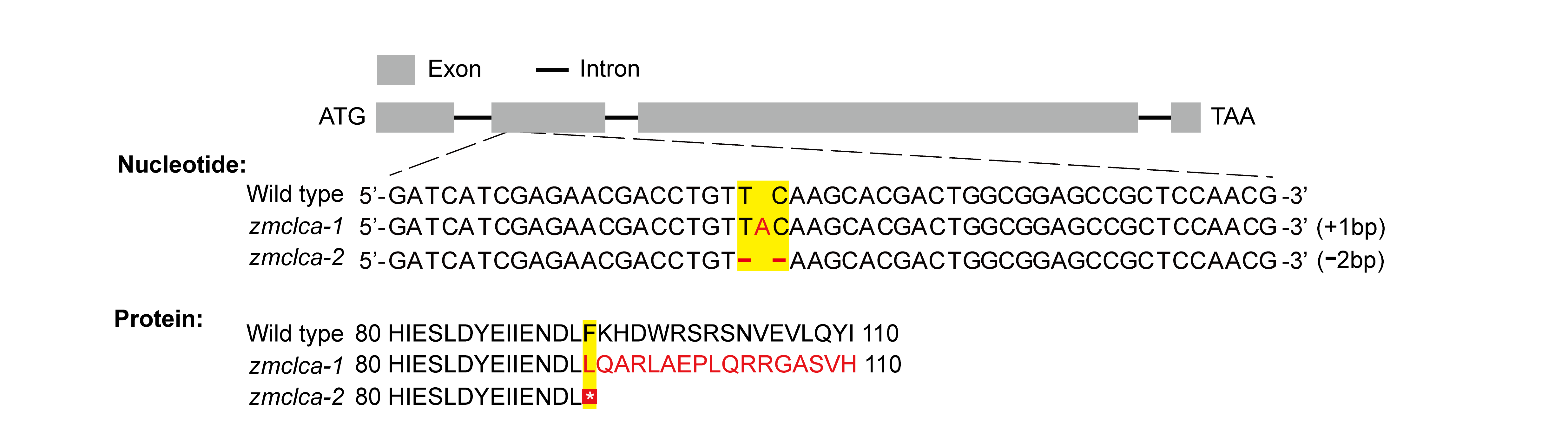


**Figure S11. Generation of *zmclca* knockout mutants.** Targeted mutagenesis of ZmCLCa using a CRISPR/Cas9-based approach. Alignment of the nucleotide sequences and the protein sequences of wild type and *zmclca* knockout mutants (*zmclca-1*, *zmclca-2*) are shown, the mutation sites are colored in red with yellow highlight, the frame-shifted sequences of *zmclca-1* are colored in red,
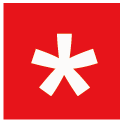
 indicates the stop codon.


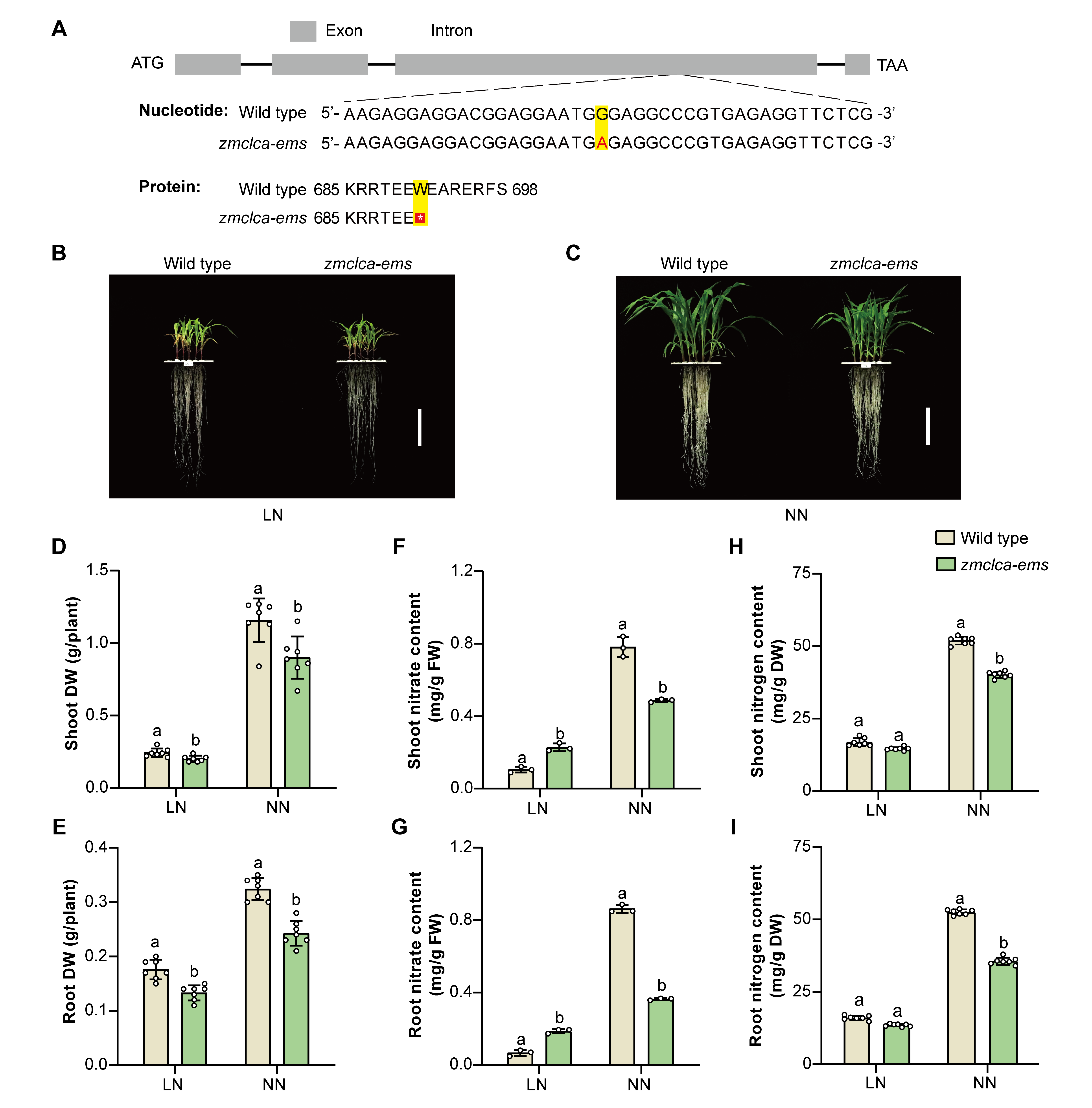


**Figure S12. ZmCLCa positively regulates maize growth and nitrate content.** (**A**) Generation of *zmclca-ems* mutant. Alignment of the nucleotide sequences and the protein sequences of wild type and *zmclca-ems* are shown, the mutation sites are colored in red with yellow highlight,
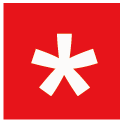
 indicates the stop codon. Three-week-old *zmclca* EMS mutant (*zmclca-ems*) and wild-type maize plants growth under (**B**) Low Nitrate (LN, 0.04 mm KNO_3_) and (**C**) Normal Nitrate (NN, 4 mm KNO_3_) conditions. (**D**, **E**) The biomass, (**F**, **G**) nitrate concentration and (**H**, **I**) nitrogen concentration in shoot and root tissue of *zmclca-ems* and wild-type maize plants grown in indicated nitrate conditions. DW, dry weight; FW, fresh weight. (**B** and **C**) Scale bars, 20 cm. Data in (**D**-**I**) are means ± SD (*n* = 3-7 biological replicates). Statistical significance was determined using two-tailed Student’s *t*-test. Different letters represent a significant difference at *p* < 0.05.


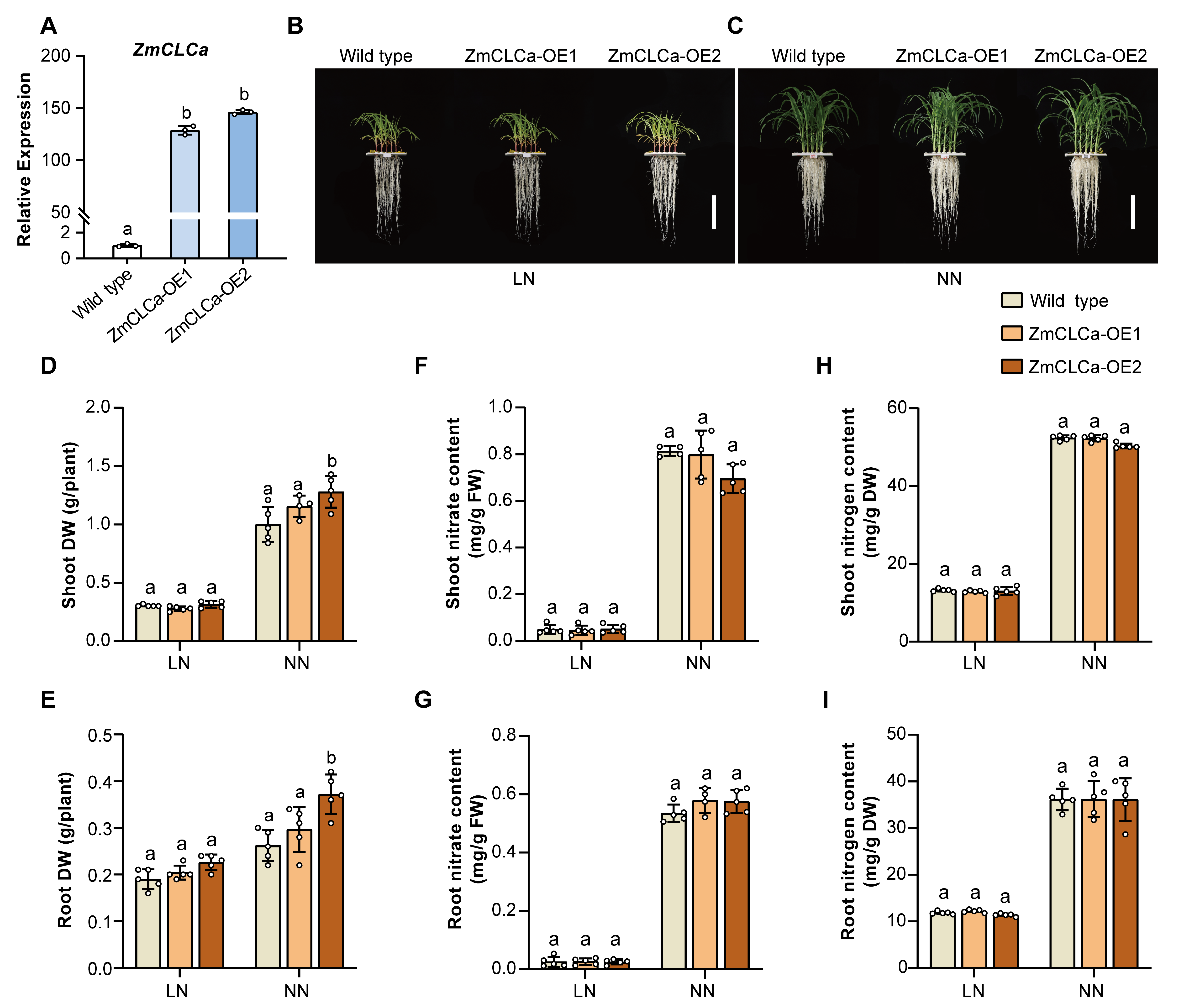


**Figure S13. Overexpression of ZmCLCa promotes maize growth.** (**A**) The transcript levels of *ZmCLCa* in wild-type and ZmCLCa overexpressing maize plants. RNA samples were prepared using entire two-leaf-old seedlings, and then the qRT-PCR assay was performed with three technical replicates. Three-week-old ZmCLCa overexpressing (ZmCLCa-OE) and wild-type maize plants growth under (**B**) Low Nitrate (LN, 0.04 mm KNO_3_) and (**C**) Normal Nitrate (NN, 4 mm KNO_3_) conditions for two weeks. (**D**, **E**) The biomass, (**F**, **G**) nitrate concentration and (**H**, **I**) nitrogen concentration in shoot and root tissue of ZmCLCa overexpressing and wild-type maize plants grown in indicated nitrate conditions. DW, dry weight; FW, fresh weight. Scale bars, 20 cm in (**B** and **C**). Data in (**D**-**I**) are means ± SD (*n* = 5 biological replicates). Statistical significance was determined using one-way ANOVA followed by Tukey’s multiple comparison test. Different letters represent a significant difference at *p* < 0.05.


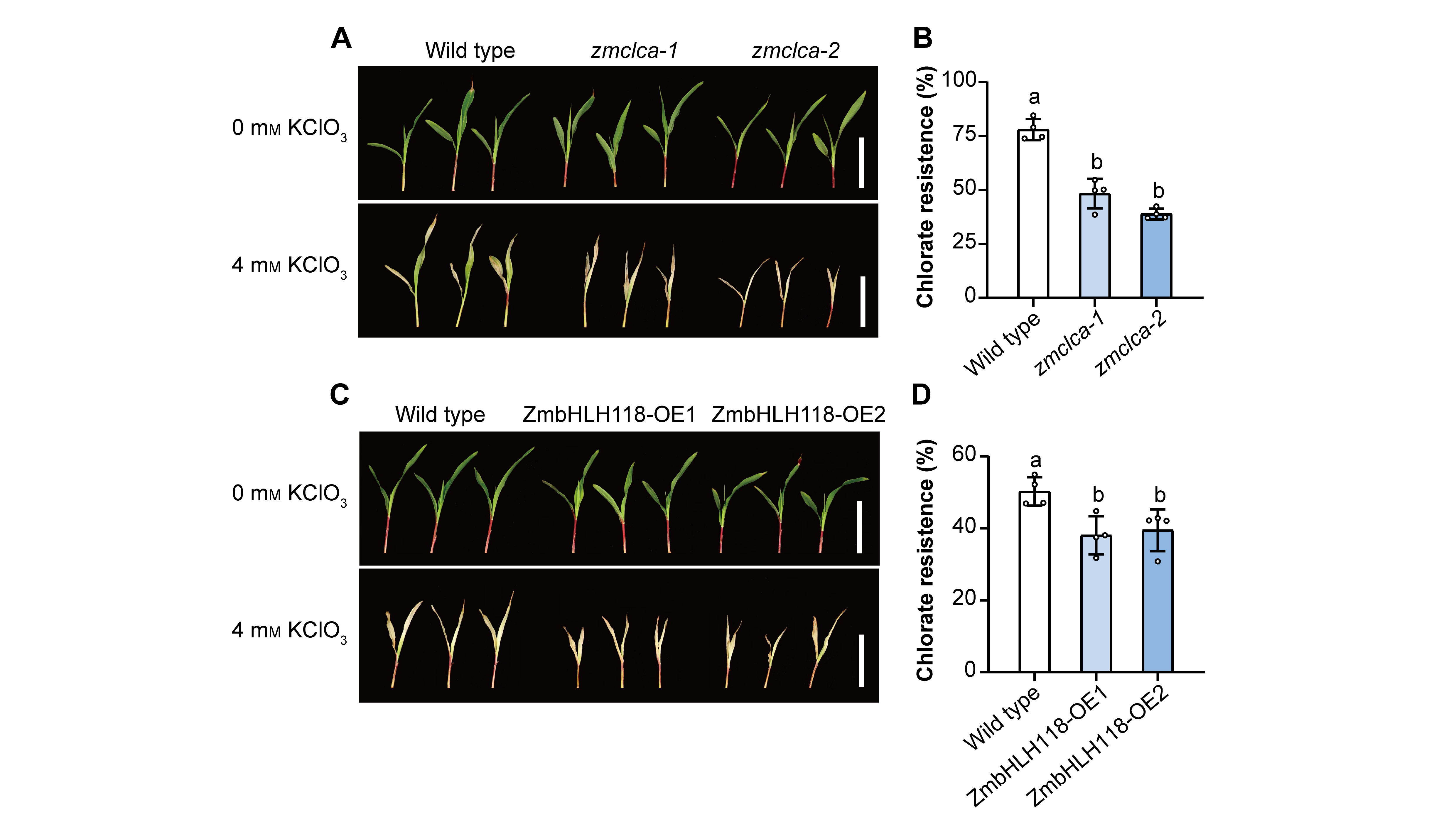


**Figure S14. ZmCLCa promotes chlorate resistance in maize.** (**A**) Growth phenotype of *zmclca* knockout mutants and wild type under different chlorate conditions (0 mm KClO_3_ and 4 mm KClO_3_) for 4 days. (**B**) Relative shoot fresh weight indicated degrees of KClO_3_ resistance. *zmclca* knockout mutant seedlings showed lower chlorate resistance. (**C)** Growth phenotype of ZmbHLH118 overexpressing seedlings and wild type under different chlorate conditions (0 mm KClO_3_ and 4 mm KClO_3_) for 5 days. (**D**) ZmbHLH118 overexpressing seedlings also showed lower chlorate resistance. Shoot fresh weight was used to evaluate chlorate resistance. Scale bars, 5 cm in (**B** and **D**). Data in (**B** and **D**) are means ± SD (*n* = 4 biological replicates). Statistical significance was determined using one-way ANOVA followed by Tukey’s multiple comparison test. Different letters represent a significant difference at *p* < 0.05.


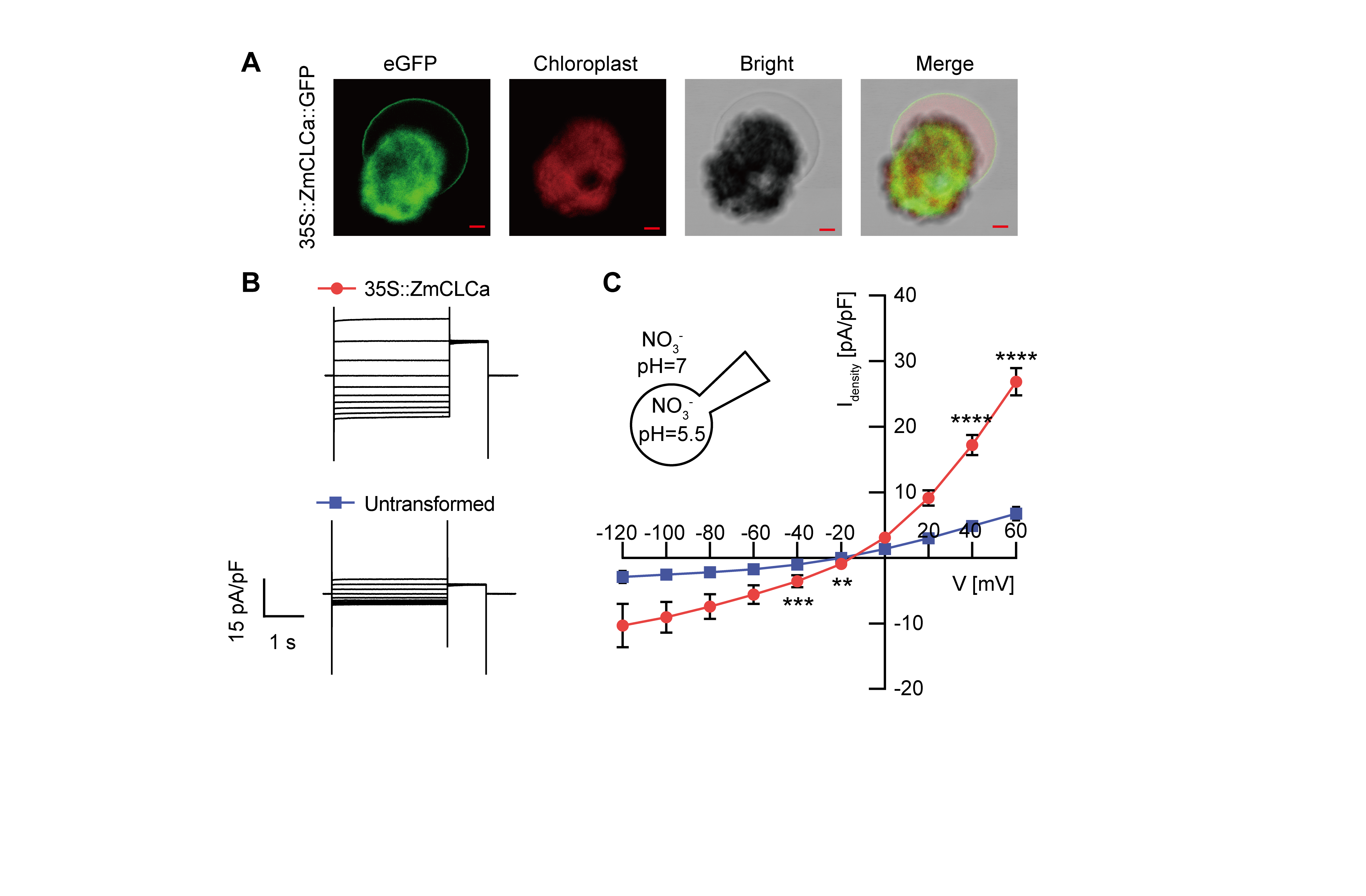


**Figure S15. ZmCLCa mediates NO_3_^-^ transport into the vacuole.** (**A**) Subcellular localization of ZmCLCa in the tonoplast of tobacco protoplast with the released vacuole. Vacuoles were released using the solution (0.1 mm CaCl_2_, 3 mm MgCl_2_, 100 mm HCl, moderate BisTrisPropane to pH 7.5, 500 mOsm). Scale bars, 10 μm. (**B**) Representative whole-vacuole currents recorded from ZmCLCa overexpressing tobacco vacuoles (upper traces) and untransformed tobacco vacuoles (lower traces). Voltage pulses were applied for 3 s from -120 to +60 mV in +20 mV increments, a post pulse of -40 mV was applied after each stimulation for 1 s. The holding potential was 0 mV. (**C**) Steady-state current density (I) from ZmCLCa and untransformed vacuoles in *Nicotiana benthamiana* in whole-vacuole configuration showed that NO_3_^-^ is transported into vacuole at slightly negative physiological membrane potentials. Cytosolic pH 7 with 4.2 mm NO_3_^-^, vacuolar pH 5.5 with 100 mm NO_3_^-^. Current densities (pA/pF) were plotted against the applied membrane potential V (mV). Data in (**C**) are means ± SD (*n* = 5 vacuoles). Statistical significance was determined using two-tailed Student’s *t*-test. **, ***, **** represent a significant difference at *p* < 0.01, *p* < 0.001, *p* < 0.0001, respectively.


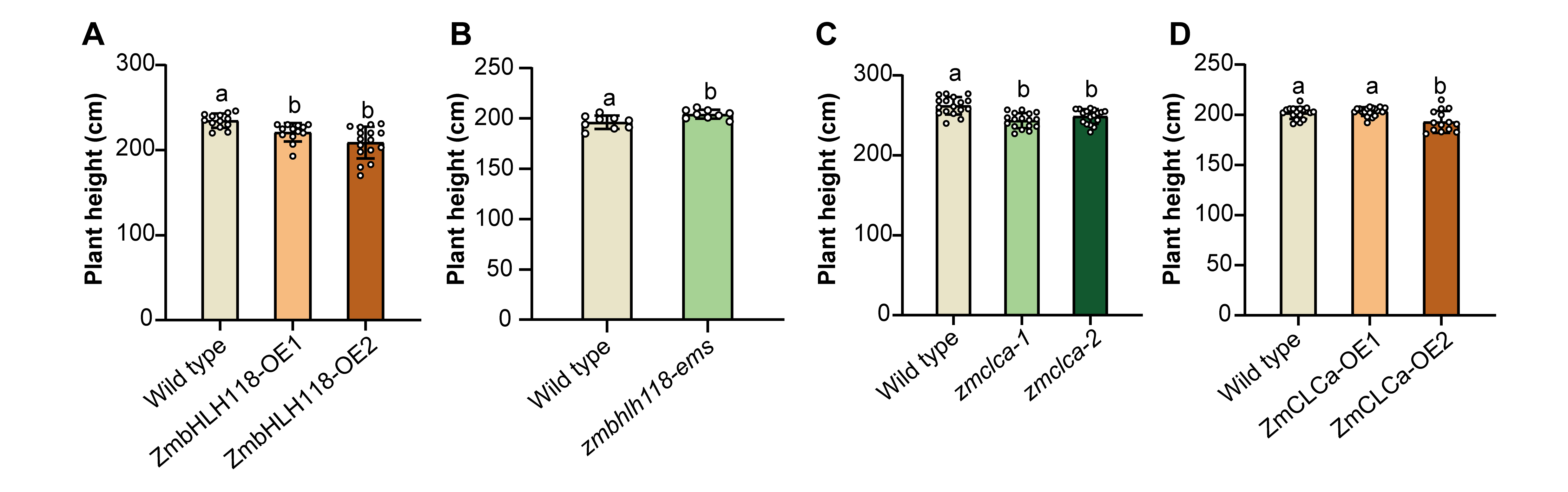


**Figure S16. Plant height of ZmbHLH118 and ZmCLCa transgenic maize plants in the field.** (**A, B**) Plant height of ZmbHLH118 overexpressing plants (**A**) and EMS mutant (**B**) at the silking stage in the filed (Sanya, Hainan). (**C, D**) Plant height of ZmCLCa knockout mutants (**C**) and overexpressing plants (**D**) at the silking stage in the filed (Shangzhuang, Beijing). Data are means ± SD (*n* ≥ 9 biological replicates). Statistical significance was determined using one-way ANOVA followed by Tukey’s multiple comparison test in (**A**, **C**, **D**) and two-tailed Student’s *t*-test in (**B**). Different letters represent a significant difference at *p* < 0.05.


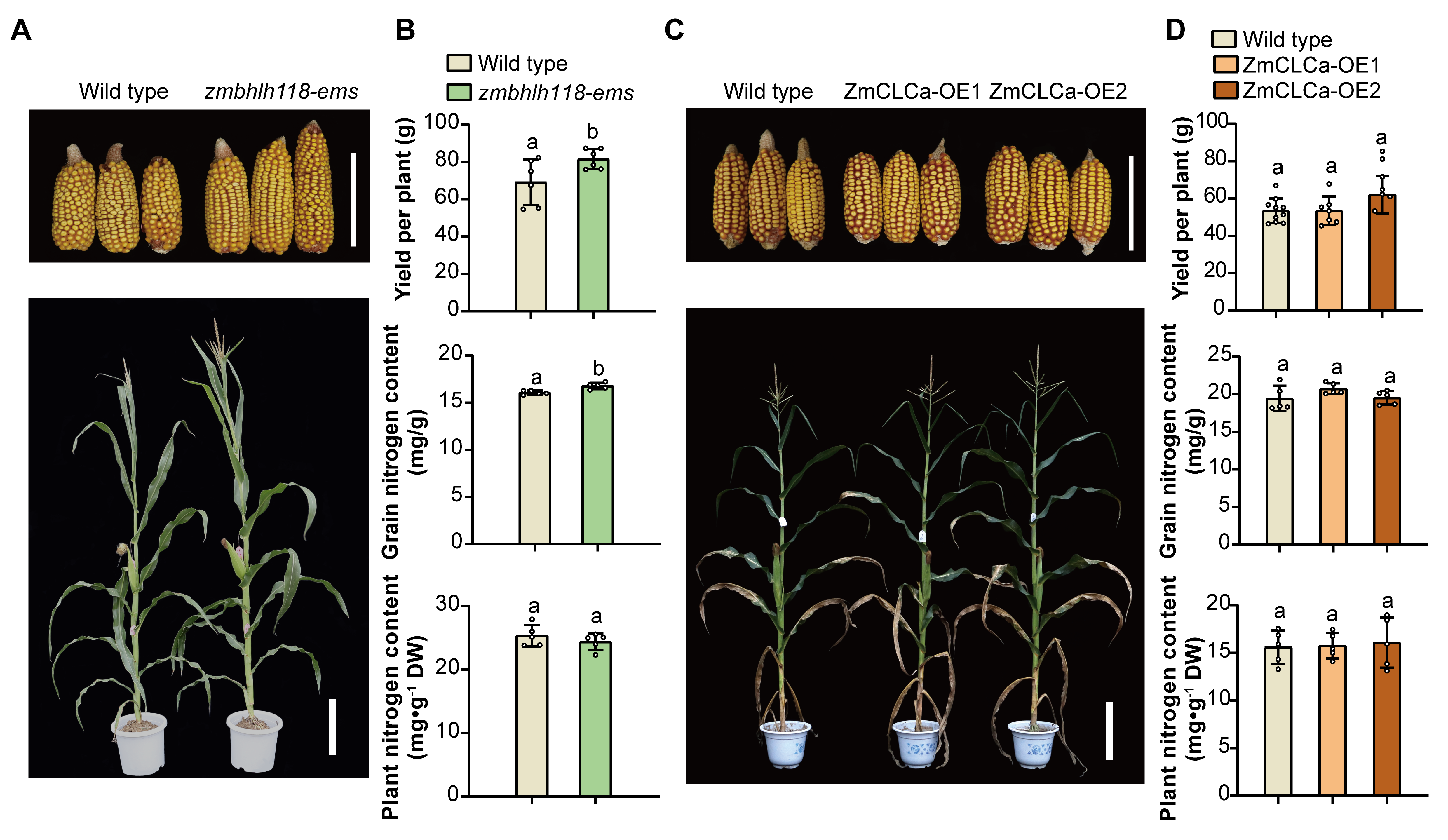


**Figure S17. Function-loss of ZmbHLH118 promotes maize growth and yield in the field.** (**A**) Representative photographs of ears (Scale bars, 10 cm) at maturation stage and plants (Scale bars, 30 cm) at silking stage of *zmbhlh118-ems* mutant (wild type: B73). (**B**) Grain yield per plant, nitrogen content of plants at the silking stage and nitrogen content of grains at the maturation stage of *zmbhlh118-ems* mutant in the filed (Sanya, Hainan). (**C**) Representative photographs of ears (Scale bars, 10 cm) at maturation stage and plants (Scale bars, 30 cm) at silking stage of ZmCLCa overexpressing plants (wild type: B104). (**D**) Grain yield per plant, nitrogen content of plants at the silking stage and nitrogen content of grains at the maturation stage of ZmCLCa overexpressing plants in the filed (Shangzhuang, Beijing). Data in (**B** and **D**) are means ± SD (*n* ≥ 5 biological replicates). Statistical significance was determined using two-tailed Student’s *t*-test in (**B**) and one-way ANOVA followed by Tukey’s multiple comparison test in (**D**). Different letters represent a significant difference at *p* < 0.05.


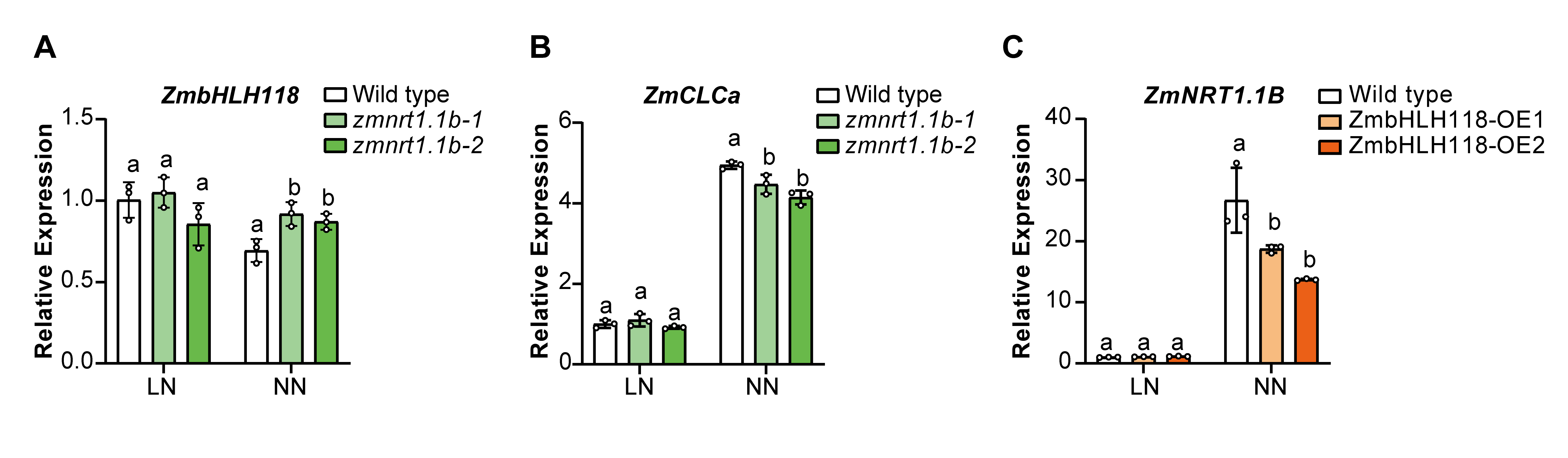


**Figure S18. The** **mutual regulation of expression levels between *ZmNRT1.1B* and *ZmbHLH118*.** The relative transcript levels of *ZmbHLH118* (**A**) and *ZmCLCa* (**B**) in root tissues of three-week-old wild type (ND101) and *zmnrt1.1b* mutants (*zmnrt1.1b-1*, *zmnrt1.1b-2*) were determined by qRT-PCR analysis. (**C**) The relative transcript levels of *ZmNRT1.1B* in root tissues of three-week-old wild-type (ND101) and ZmbHLH118 overexpressing maize plants (ZmbHLH118-OE1, ZmbHLH118-OE2) were determined by qRT-PCR analysis. *ZmTUB* was used as the internal reference and the expression level in the wild type under LN condition was set to 1. Data in (**A-C**) are means ± SD (*n* = 3 technical replicates). Statistical significance was determined using one-way ANOVA followed by Tukey’s multiple comparison test. Different letters represent a significant difference at *p* < 0.05.


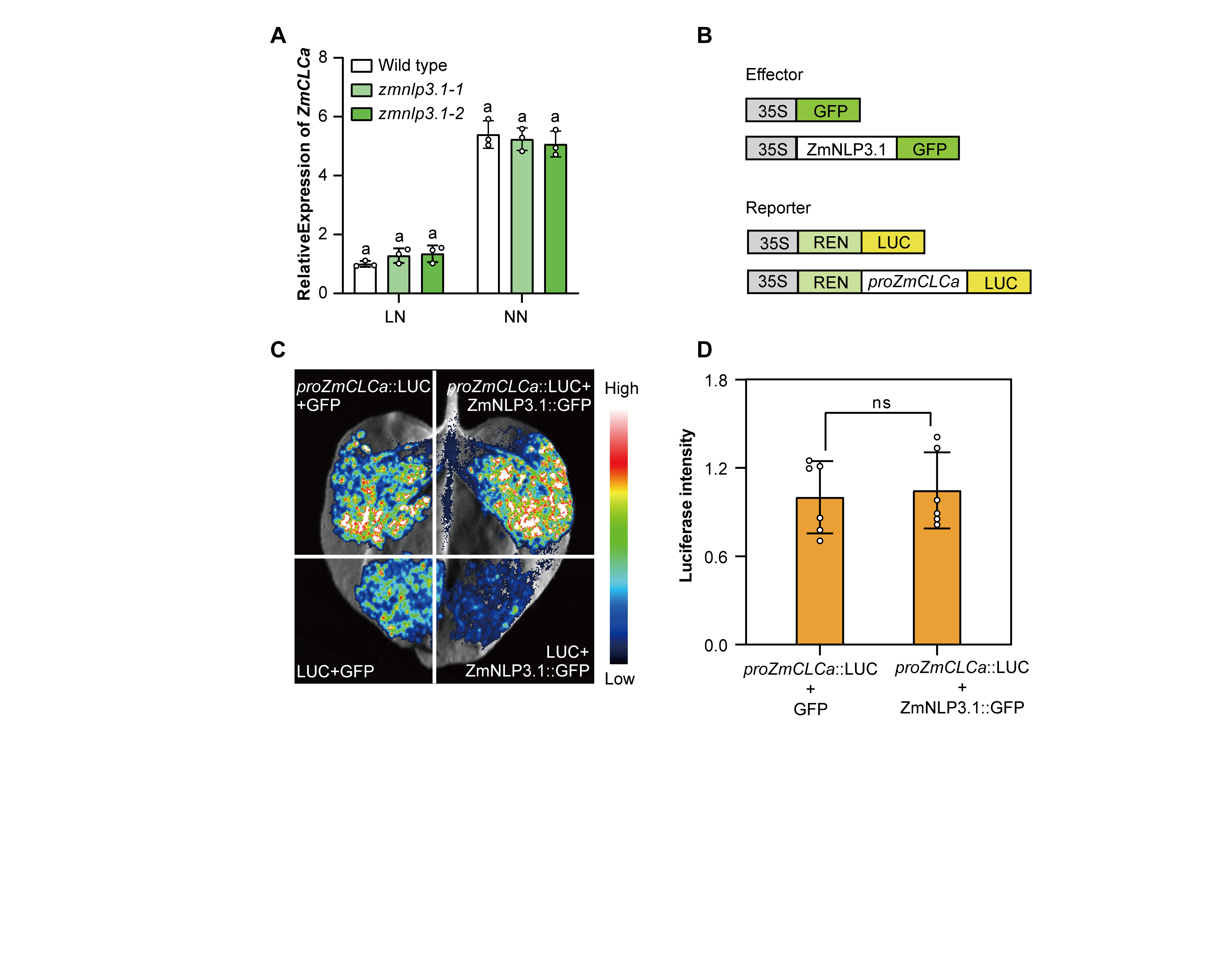


**Figure S19. ZmNLP3.1 does not regulate *ZmCLCa* expression.** (**A**) The relative transcript levels of *ZmCLCa* in root tissues of three-week-old wild type and *zmnlp3.1* mutants (*zmnlp3.1-1*, *zmnlp3.1-2*) were determined by qRT-PCR analysis. *ZmTUB* was used as the internal reference and the expression level in the wild type under LN condition was set to 1. Data in (**A**) are means ± SD (*n* = 3 technical replicates). Statistical significance was determined using one-way ANOVA followed by Tukey’s multiple comparison test. Different letters represent a significant difference at *p* < 0.05. (**B**) Schematic diagram of the reporter and effector constructs used in the dual luciferase assay system. (**C**) The appearance of dual luciferase assay and (**D**) the relative luciferase intensity of the *ZmCLCa* promoter activity co-expressing with ZmNLP3.1 in *Nicotiana benthamiana*. The luciferase intensity was calculated by ImageJ of six biological replicates. The luciferase intensity of *proZmCLCa*::LUC + GFP was set to 1. Data in (**D**) are means ± SD (*n* = 6 biological replicates). Statistical significance was determined using two-tailed Student’s *t*-test. ns represents no significant difference (*p* > 0.05).

**Table S1. List of primers used in this study.**

| Primer name | Primer sequence (5’-3’) |
| --- | --- |
| **Primers for quantitative real-time PCR.** | |
| ZmTUB-F | GCTATCCTGTGATCTGCCCTGA |
| ZmTUB-R | CGCCAAACTTAATAACCCAGTA |
| ZmbHLH118-F | TTGGAGCAGATGCTCAAGGG |
| ZmbHLH118-R | GGACGAACTTTCCAGCCTGA |
| ZmCLCa-F | GGACAGCAAGAACGACGACAG |
| ZmCLCa-R | CATGGCCCACTTGAGGAAGAT |
| ZmCLCc-F | GATCTTAGCGCCCAGTACCC |
| ZmCLCc-R | AGAACCGGTGTGCACCATAG |
| ZmCLCd-F | CACCTGGCACAGATGGGAAT |
| ZmCLCd-R | TATTCCGGGAACGAACTGGC |
| ZmCLCe-F | GGTGTATGCAAGGTGCCTCT |
| ZmCLCe-R | AGCATGTCGTTTGCTTGCTG |
| ZmCLCf-F | GTCTTATGGAGGCCAGAGGG |
| ZmCLCf-R | CTATTTCCTCCCTTGACGAGCA |
| ZmCLCg1-F | GAACGACGTGTTCAAGCAGG |
| ZmCLCg1-R | GACGGGAACTGGTACCTTCA |
| ZmCLCg2-F | CCATCGTGGTACACTGCAAG |
| ZmCLCg2-R | AACACGGTGCTGACATCGTA |
| ZmNRT1.1B-F | TGCTGCTGCCAGTGCCACAA |
| ZmNRT1.1B-R | ACACGTTAATTAGCTCGACCTGCG |
| ZmNRT2.1-F | CGGAGCATGTCTAAGTCT |
| ZmNRT2.1-R | GCACAGTAATAACAAGGCATA |
| ZmNR1.1-F | GATCAAAGGATACGCATACT |
| ZmNR1.1-R | TCGACGTCGACGGACCAGAA |
| ZmNIR1.1-F | CTGGACCGGATGCCCCAACA |
| ZmNIR1.1-R | CGACGCGGCCGCCCACGAAG |
| **Primers for genotyping.** | |
| ZmbHLH118-EMS-F | TCAACGAGCTCTGTGCCATC |
| ZmbHLH118-EMS-R | CTTGGTCGTGTCGAGGGAC |
| ZmCLCa-CRISPR-F | CGGATTCGGACAGCAAGAAC |
| ZmCLCa-CRISPR-R | GCTTGACCTTGACGATGAGC |
| ZmCLCa-EMS-F | CCATCATCCTCATGGGCTCC |
| ZmCLCa-EMS-R | GAGGGAATGCACCAAGGATGT |
| **Primers for localization assay.** | |
| ZmbHLH118-GFP-F | gacgagctcggtaccATGAGCTGCGCGGGGC |
| ZmbHLH118-GFP-R | TTTGCCCATGTCGACGGCGACCGGCGGCC |
| ZmCLCa-GFP-F | gacgagctcggtaccATGGAGGAAGAGCAGAGCCC |
| ZmCLCa-GFP-R | TTTGCCCATgtcgacGTGTACTTTCCTTTTGTTTGC |
| **Primers for Dual-LUC.** | |
| Pro-ZmCLCa-F | TACGAATTCGAGCTCGTTAAGTGCACCTTGACACCTAAC |
| Pro-ZmCLCa-R | GACTCTAGAGGATCCGGCTTTCCCCGGGACC |
| Super-ZmbHLH118-F | gacgagctcggtaccATGAGCTGCGCGGGGC |
| Super-ZmbHLH118-R | TTTGCCCATgtcgacGGCGACCGGCGGCC |
| Super-ZmbHLH162-F | GACGAGCTCGGTACCATGTCTCTCGCCCCGGAC |
| Super-ZmbHLH162-R | TTTGCCCATGTCGACTGCAACAGGAGGGCACGC |
| Super-ZmbHLH164-F | GACGAGCTCGGTACCATGGCCTCCCCCGAGG |
| Super-ZmbHLH164-R | TTTGCCCATGTCGACCGCCACAGGAGGACAAGACC |
| Super-ZmbHLH172-F | GACGAGCTCGGTACCATGTCTCTCCCCCCTGGCC |
| Super-ZmbHLH172-R | TTTGCCCATGTCGACTGCGACAGGAGGGCACG |
| Super-ZmNLP3.1-F | GACGAGCTCGGTACCATGGACTTCGACCCCTCC |
| Super-ZmNLP3.1-R | TTTGCCCATGTCGACACCAGAGCTTCCACAAGAAC |
| **Primers for Y1H.** | |
| AD-ZmbHLH118-F | GAGGCCAGTGAATTCATGAGCTGCGCGGGGC |
| AD-ZmbHLH118-R | CTGCAGCTCGAGCTCCTAGGCGACCGGCGGC |
| BD-Pro ZmCLCa-F | cttgaattcgagctcTGTTGTAGCAGGGAGCAAATAATGC |
| BD-Pro ZmCLCa-R | agatccccgggtaccGGCTTTCCCCGGGACC |
| BD-Pro P1-F | cttgaattcgagctcCAACCTACGAATCATAGTCCGGTG |
| BD-Pro P1-R | agatccccgggtaccGCACCTACCATTTTGTGAAGAAGC |
| BD-Pro P2-F | cttgaattcgagctcGTTCGTGGTTCATCGAACTAGG |
| BD-Pro P2-R | agatccccgggtaccTCTATGGATGGACTGGCGGAC |
| BD-Pro P3-F | cttgaattcgagctcCAATCCATCCATAGAGGCAAGG |
| BD-Pro P3-R | agatccccgggtaccGGTTCACAGCTCGCACTTC |
| BD-Pro P4-F | cttgaattcgagctcTGGTGACCTCCCAAGAGGC |
| BD-Pro P4-R | agatccccgggtaccCGTGATGAAGAAAATTCAGCACAG |
| BD-Pro P5-F | cttgaattcgagctcACAAAGCACATTCTGAAAGC |
| BD-Pro P5-R | agatccccgggtaccCAGACACCTCAACTTCATATAGGCC |
| BD-Pro P6-F | cttgaattcgagctcGCTGCCTCTGTCAATGGGTC |
| BD-Pro P6-R | agatccccgggtaccGGCTTTCCCCGGGACC |
| **Primers for ChIP-qpcr.** |  |
| Puc19-ZmbHLH118-F | gacgagctcggtaccATGAGCTGCGCGGGGC |
| Puc19-ZmbHLH118-R | TTTGCCCATGTCGACGGCGACCGGCGGCC |
| ChIP-Pro P1-F | CAACCTACGAATCATAGTCCGGTG |
| ChIP-Pro P1-R | GCACCTACCATTTTGTGAAGAAGC |
| ChIP-Pro P2-F | GTTCGTGGTTCATCGAACTAGG |
| ChIP-Pro P2-R | TCTATGGATGGACTGGCGGAC |
| ChIP-Pro P3-F | CAATCCATCCATAGAGGCAAGG |
| ChIP-Pro P3-R | GGTTCACAGCTCGCACTTC |
| ChIP-Pro P4-F | TGGTGACCTCCCAAGAGGC |
| ChIP-Pro P4-R | CGTGATGAAGAAAATTCAGCACAG |
| ChIP-Pro P5-F | ACAAAGCACATTCTGAAAGC |
| ChIP-Pro P5-R | CAGACACCTCAACTTCATATAGGCC |
| ChIP-Pro P6-F | GCTGCCTCTGTCAATGGGTC |
| ChIP-Pro P6-R | GGCTTTCCCCGGGACCGG |
| ChIP-Pro P7-F | AAAAGACGCGAGAGAAGGCC |
| ChIP-Pro P7-R | CTGGCTGGACCGACAACTTG |
| **Primers for EMSA.** |  |
| EMSA-Probe 3a-F | TGAGGTTGCCAAGGGGGCAAGTGCGTGGTCACAAAAGTGA |
| EMSA-Probe 3a-R | TCACTTTTGTGACCACGCACTTGCCCCCTTGGCAACCTCA |
| EMSA-Probe 3b-F | GTGTCCATTGCACTCTTCACCTGAAGTGCGAGCTGTGAAC |
| EMSA-Probe 3b-R | GTTCACAGCTCGCACTTCAGGTGAAGAGTGCAATGGACAC |
